# Roadmap on Digital Holography-Based Quantitative Phase Imaging

**DOI:** 10.3390/jimaging7120252

**Published:** 2021-11-26

**Authors:** Vinoth Balasubramani, Małgorzata Kujawińska, Cédric Allier, Vijayakumar Anand, Chau-Jern Cheng, Christian Depeursinge, Nathaniel Hai, Saulius Juodkazis, Jeroen Kalkman, Arkadiusz Kuś, Moosung Lee, Pierre J. Magistretti, Pierre Marquet, Soon Hock Ng, Joseph Rosen, Yong Keun Park, Michał Ziemczonok

**Affiliations:** 1 Division of Biological and Environmental Sciences and Engineering, King Adullah University of Science and Technology, Thuwal 23955-6900, Saudi Arabia; Christian.Depeursinge@kaust.edu.sa (C.D.); Pierre.Magistretti@kaust.edu.sa (P.J.M.); 2 Institute of Electro-Optical Engineering, National Taiwan Normal University, Taipei 11677, Taiwan; 3 Institute of Micromechanics and Photonics, Warsaw University of Technology, 02-525 Warsaw, Poland; arkadiusz.kus@pw.edu.pl (A.K.); michal.ziemczonok.dokt@pw.edu.pl (M.Z.); 4 Univ. Grenoble Alpes, CEA, LETI, DTBS, 38000 Grenoble, France; cedric.allier@cea.fr; 5 Optical Sciences Centre and ARC Training Centre in Surface Engineering for Advanced Materials (SEAM), School of Science, Swinburne University of Technology, Hawthorn 3122, Australia; vanand@swin.edu.au (V.A.); sjuodkazis@swin.edu.au (S.J.); soonhockng@swin.edu.au (S.H.N.); 6 School of Electrical and Computer Engineering, Ben-Gurion University of the Negev, P.O. Box 653, Beer-Sheva 8410501, Israel; mirilasn@post.bgu.ac.il (N.H.); rosenj@bgu.ac.il (J.R.); 7 Tokyo Tech World Research Hub Initiative (WRHI), School of Materials and Chemical Technology, Tokyo Institute of Technology, 2-12-1, Ookayama, Meguro-ku, Tokyo 152-8550, Japan; 8 Department of Imaging Physics, TU Delft, Lorentzweg 1, 2628 CN Delft, The Netherlands; j.kalkman@tudelft.nl; 9 Department of Physics, Korea Advanced Institute of Science and Technology (KAIST), Daejeon 34141, Korea; lkaamo@kaist.ac.kr (M.L.); yk.park@kaist.ac.kr (Y.K.P.); 10 KAIST Institute for Health Science and Technology, KAIST, Daejeon 34141, Korea; 11 CERVO Brain Research Center, CIUSSS de la Capitale-Nationale, Québec, QC G1E 1T2, Canada; pierre.marquet@neuro.ulaval.ca; 12 Joint International Research Unit, Université Laval, Québec, QC G1V 0A6, Canada; 13 Tomocube Inc., Daejeon 34109, Korea

**Keywords:** quantitative phase imaging, digital holographic microscopy, holographic tomography, 3D distribution of refractive index, biomedical analysis at cellular level

## Abstract

Quantitative Phase Imaging (QPI) provides unique means for the imaging of biological or technical microstructures, merging beneficial features identified with microscopy, interferometry, holography, and numerical computations. This roadmap article reviews several digital holography-based QPI approaches developed by prominent research groups. It also briefly discusses the present and future perspectives of 2D and 3D QPI research based on digital holographic microscopy, holographic tomography, and their applications.

## 1. Introduction

Quantitative Phase Imaging (QPI) refers to a number of label-free microscopy techniques that provide contrast by quantifying the phase changes in the wavefront when light propagates through a transparent specimen [1,2]. QPI provides unique means for imaging biological or technical microstructures, merging beneficial features identified with microscopy, interferometry, holography, and numerical computations. In biomedical applications, QPI (using refractive index as the endogenous contrast agent) numerically converts recorded interference pattern into a nanoscale-precise subcellular-specific map of optical delay introduced by the examined specimen [3,4,5].

The various technical approaches available to achieve QPI can be categorized into two groups: first, QPI solutions based on digital holographic microscopy (DHM) [3], holographic tomography (HT) [6,7], spatial interference interferometry (SLIM) [8], gradient light interference microscopy (GLIM) [9], quadriwave lateral shearing interferometry (QLSI) [10], Hilbert phase microscopy (HPM) [11], and Hilbert–Huang phase microscopy (H2PM)[12], and secondly, QPI-based on iterative multi-frame phase retrieval i.e., Fourier ptychography [13,14] or transport of intensity [15].

As mentioned above, QPI is a very broad topic. The most popular and widely commercialized QPI techniques are those based on digital holography, namely: DHM (also referred in literature as quantitative phase microscopy, 2D QPI) and HT (also referred in literature as: optical diffraction tomography (ODT), tomographic diffractive microscopy (TDM), synthetic aperture microscopy (SAM), phase nanoscopy, tomographic phase microscopy (TPM), refractive index tomography (RIT), optical diffraction microscopy (ODM), and 3D quantitative phase imaging (3D QPI)). In this roadmap article, we focus mainly on providing insight and discussing the present and future trends of these two holography based QPI techniques. However, several approaches and concepts presented below can easily be extended to other QPI techniques. However, as in any overview article of this nature, it is not possible to represent all the possible approaches, applications and trends in the field of QPI. We apologize in advance if we have not included any relevant work in QPI.

This roadmap begins with the applications of digital holography to microscopy in biology by C. Depeursinge and P. Marquet (Section 2). It provides an overview and discusses the benefits of DHM. They briefly describe the label-free live cell imaging with digital holography and discuss coherent noise issues and the biological interpretation of quantitative phase signals. Next, C. Depeursinge and P. Magistretti (Section 3) introduce three-dimensional (3D) holographic microscopic techniques, provided by holographic tomography, and present their biological applications. N. Hai and J. Rosen (Section 4) discuss the implementation of QPI by self-reference on-axis holography, and they also compare the performance of multiple-shot approach with iterative, single-shot method. QPI applications in material science, especially tracking the ultra-fast material transformation with permittivity transients, is provided by S. Juodkazis, et al. in Section 5.

Three-dimensional QPI, and its extensions in estimating the refractive index profiles, are elaborated by M. Lee and Y. Park (Section 6). This section also discusses challenges referring to the image quality, imaging throughput, and data size, as well as interpretation of 3D data. V. Balasubramani and C. J Cheng (Section 7) introduce an integrated dual-mode tomography approach, which addresses the missing cone problem and provides an enlarged spatial frequency coverage, resulting in high-quality image reconstruction. J. Kalkman (Section 8) describes the challenges connected with the implementation of ultra-scale (i.e., with optimized throughput) and high-contrast 3D QPI, which are needed to open up new biomedical applications. M. Kujawinska, et al. (Section 9) discuss the importance of metrology aspects in 3D QPI, including the proper determination of accuracy, precision of instruments, and uncertainty of the measurements, as well as the need of standardized 3D phantom. C. Allier (Section 10) discusses the evolution from computational to neural microscopy and the importance of deep learning solutions, which can replace conventional algorithms in 2D and 3D phase microscopy. This section also shows the design of the neural microscopy framework for QPI. Finally, Section 11 summarizes the presented work and draws conclusions.

The authors believe that this roadmap article will be of interest to the young scientists and researchers who are working in the field or plan to explore and further develop different aspects of the quantitative phase imaging techniques and their applications.

This section was prepared by Malgorzata Kujawinska and Vinoth Balasubramani.

## 2. Application of Holography to Microscopy in Biology: Label-Free Live Cell Imaging with Digital Holographic Microscopy

### 2.1. Status

Optical microscopes are one of the most productive scientific instruments. Nevertheless, the limits on resolution, formulated by the well-known Abbe law, as well as the lack of quantitative data, have appeared as severe limitations. Holography [16], as a means to reconstruct the specimen with its 3D shape, together with its dielectric properties, was a breakthrough. In microscopy, the theoretical basis was given by E. Wolf [17]. The adaptation of digital holography to microscopy soon revolutionized the domain. In the 1990s, the team directed by one of us chose to develop the application of Digital Holography (DH) to micro-endoscopy and microscopy. The first quantitative phase image of a living neuron was obtained by Etienne Cuche in 1999 [18] as well as Pierre Marquet [19]. The determination of the precise topology (with nanometer accuracy), together with Refractive Index (RI) and polarizability [20,21,22], were henceforth possible by DHM. Finally, a major asset of holography is that complex waves, scattered by the specimen, could be determined from a single hologram acquired in a snapshot, thereby avoiding motion blur and chaotic movement effects.

**Figure 1:**
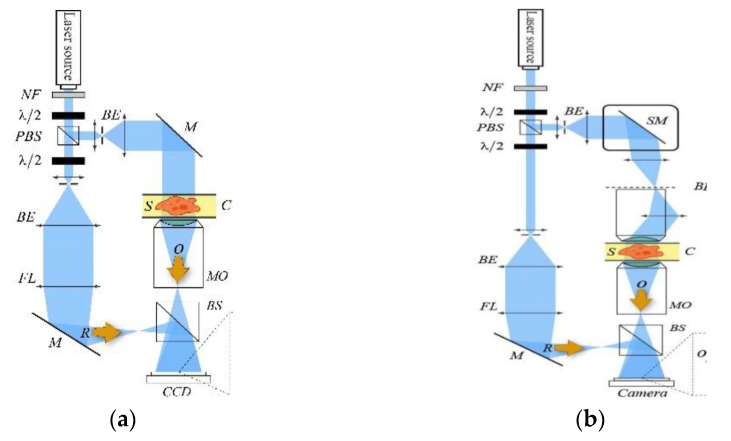
(**a**) example of optical setup for transmission Digital Holographic Microscopy DHM. (**b**) Optical setup for Tomographic Diffractive Microscopy: Laser source with controllable coherence length. *NF* neutral filter, λ/2 plate, *PBS* polarizing beam splitter, *BS* beam splitter, *BE* beam expander, *SM* steering mirror and *M* mirror, BF back focal plane, *S* specimen, *C* cell, *O* object wave, R reference wave.

The principle of DHM applied to biological objects is given in Figure 1. On the right part of the sketch, one can recognize the optical scheme of a traditional microscope in transmission. On the left, an optical path is added in order to interfere with the beam diffracted by the specimen. The camera then captures a hologram that forms the image of the specimen after reconstruction by digital means. For more details, refer to [23].

QPI has developed dramatically over the last 20 years, thanks in particular to both the availability of inexpensive digital image sensors with a high pixel number of small size (between ~1 μm and ~10 μm) and the increase in computing power allowing us to process digital images of several megapixels. The approaches based on DH occupy an important place in QPI, particularly because they are generally robust and simple to implement. Moreover, the numerical reconstruction of the digitally recorded holograms makes it possible to obtain the whole complex diffracted wavefront, i.e., both its amplitude and phase, thus offering the possibility of propagating it to different planes. Such numerical propagation provides many advantages, including the ability to perform autofocusing, extended depth of focus, and to correct any kind of aberration, especially those introduced by a microscope objective. Thus, high-resolution extended-depth-of-field quantitative phase images, well suited for live cell imaging, can be obtained with DHM. Knowing that most biological cells are transparent, the phase information of the scattered wavefront represents an intrinsic contrast to visualize them non-invasively without any staining. As far as weakly diffracting specimens are concerned, such as living cells, the phase retardation of the transmitted light wave, namely quantitative phase signal (QPS), is given by the following equation,

(1) QPS=2πλ(nc−nm)d 

where *n_m_* is the RI of the surrounding medium, *n_c_*, and *d*, the RI averaged over the corresponding optical path length and the thickness of the observed specimen, respectively. QPS, stemming from the difference between *n_c_* and *n_m_*, has allowed the development of several very appealing applications, including cell culture inspection [24], automated cell counting, recognition, and classification for diagnostic purposes [25], with the use of machine learning approaches [26]. In addition, promising applications aiming at identifying neoplastic lesions from histologic samples [27], assessing cellular responses induced by new drugs [28], and performing label-free high content screening [29] were successfully carried out. Furthermore, it is well known, [30] that a cell’s dry mass (DM) can be extracted from QPS, and its monitoring is especially permitted to characterize cell behavior in response to stress [31], cell cycle [32], and mass transport in cultured neuronal networks [33]. The ability of numerical propagation to apply extended depth of focus has made efficient semen analyses possible, which represents appealing developments in fertility medicine [34].

### 2.2. Current and Future Challenges

Despite these very attractive applications, the QPS is often affected by coherent noise (1) and is not very specific to interpret in terms of cellular processes (2). This limits the capacity of QPS to study significant questions in cell biology.

### 2.3. Advances in Science and Technology to Meet Challenges

**Figure 2:**
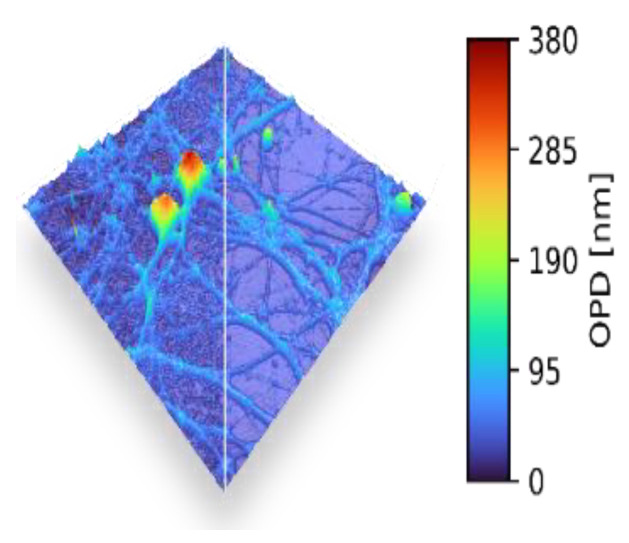
3D representation of color-coded optical path difference (OPD) of a living neuronal network. The right part of the image is quasi speckle-free thanks to a polychromatic DHM approach [37], allowing to study neuronal processes and network connectivity.

Noise issue: It is well known that the light source coherence, useful to generate high-quality interference patterns, that encode the phase in an extremely accurate manner, generates coherent noise (CN) that significantly degrades the quantitative phase image quality. Some strategies, based on either numerical approaches [35] or optical instrumentation developments [36,37], have started to be able to significantly decrease CN (Figure 2).

Biological interpretation of QPS: QPS is very valuable since it contains information about both cell content and morphology through the parameters *n_c_* and *d*, respectively, (see Equation (1)), which enables the monitoring of certain important processes, including the cell cycle [38]. Unfortunately, this dual information is mixed together, which can often make the QPS interpretation elusive. One way to overcome these limitations is to have a good knowledge of the cell system studied in order to make assumptions about the behavior of *d* or *n_c_*. For example, under an assumption of *n_c_* invariance, studies of red blood cell (RBC) membrane fluctuations, conducted at a nanometric scale with DHM, or of cell deformation induced by shear forces, lead to the detection of highly relevant biomechanical parameters as possible biomarkers of diseases [39,40]. Several attempts have been proposed to obtain *d* and/or *n_c_* separately [40,41,42,43,44]. The methodology presented in [44], based on a perturbation of the extracellular RI, has the main advantage of providing, without making any assumption about the shape of the cell or exerting any constraint on it, simultaneous measurements of the spatial average cell RI ${\bar{n}}_{c}$, thickness $\bar{d}$, and the absolute cell volume *Vc*, with accuracies of 0.0006, 100 nm, and 50 µm^3^, respectively. *Vc* is an essential cellular parameter, finely regulated, which, however, remains poorly studied because of the difficulty to measure it. Measuring ${\bar{n}}_{c}$ and $\bar{d}$ separately represents a key step, leading to the calculation of various relevant cellular biophysical parameters, including, in addition to *Vc*, the osmotic membrane water permeability, the RI of transmembrane water and solute flux [21], DM concentration, as well as the mean corpuscular volume and hemoglobin concentration of RBCs [45]. This perturbation approach [43] only allows the measurement of relatively slow volume changes (0.1 Hz) and provides coarse information on the local changes in cell morphometry. Within this framework, holographic tomography approaches [7] present the great advantage of providing the full 3D distribution of the intracellular RI $n_{c}\left(r\right)$. Some approaches of tomographic phase microscopy, as presented in this section, enable ways to significantly increase the resolution. Such 3D $n_{c}\left(r\right)$ distribution offers the very attractive possibility to visualize some specific organelles inside the cell, including the nucleus, mitochondria, and lipid droplets. However, the different experimental tomographic set-ups remain demanding from an optomechanical point of view, and the reconstruction algorithms are complex and time consuming for obtaining $n_{c}\left(r\right)$ with a good accuracy [46].

### 2.4. Concluding Remarks

QPI techniques, in general, and DHM, in particular, allow a non-invasive visualization of living cells without the use of contrast agents. However, to address important questions related to cell biology and diseases, future developments are needed to exploit the richness of the QPS in order to measure important biophysical cell parameters, such as absolute cell volume.

This section was prepared by Pierre Marquet and Christian Depeursinge.

## 3. Principle and Application of Tomographic Phase Microscopy in Biology

### 3.1. Tomographic Phase Microscopy

In general, a wavefront determination obtained from a single hologram does not suffice to obtain full 3D imaging of an object because the aperture of the hologram is limited by both the magnification optics and, potentially, by the limited resolution of the camera. This is true for all imaging modalities, based on holography or not. The main benefit of holography is that it provides an easy way to combine the complex wavefields by simple addition of complex numbers: digital holography provides, by reconstruction, a complete characterization of the wavefront propagating in the dielectric medium. The wave is described by a complex number involving its amplitude and phase. It is governed by a linear equation: the Helmholtz equation. The solution of the propagation equation can be sought in Fourier space. Then, the Fourier components of the diffraction potential can be deduced from the distribution of the complex field, provided by holographic reconstruction, on the Ewald sphere and for a particular direction of the incident wave. In order to have all the Fourier components of the diffraction potential and, therefore, the exact distribution, in 3D space, of the index of refraction, the specimen should be irradiated from different angles of incidence and the complex values collected on the corresponding Ewald spheres. In the end, these complex values can be simply added in this context. The microscope objectives (MO) aberrations can also be analyzed in detail and introduced in the numerical model of wave propagation [47]. Complex deconvolution is a direct benefit of these developments. In the frequency domain, it is feasible to compensate for chromatic aberrations to achieve perfect superposition of wavefronts reconstructed from digital holograms at different wavelengths. Similarly, the adjustment of fields in 3D space and, subsequently, in the wavevector space can be achieved precisely. The concept of synthetic aperture has been developed [48,49,50] in our group, and it has been developed both in the time and in the spatial domain: it derives directly from the considerations developed in relation to Abbe’s theory of the resolution limit caused by the necessarily limited aperture of the physical microscope. This limitation can be overcome if one can extend, in Fourier space, the determination of the Fourier components of the diffusion potential. This is possible either by taking the data at various wavelengths of the irradiating field (time/frequency domain) or at various incidence angles of the irradiating wave (spatial/K-space domain). This procedure makes it possible to artificially increase the aperture of the microscope; for this reason, we speak of synthetic aperture. The first variant of this approach consists in changing the wavelength and varying, accordingly, the amplitude of the wavevector in K-space. Multiple wavelengths have been used to reconstruct 3D structures [51,52,53]. The wavelength scan is quite small, accordingly limiting the resolution. A somewhat similar approach is to use a partially coherent source to form a hologram in the plane where the mutual coherence between object and reference wave is non-zero: this concept introduces coherence gating in the spatial domain, which proved to perform well [54,55,56]. The second variant of this approach consists of varying the angle of K-vector and the illumination waves—variable direction—and can be used in conjunction with the previous technique where the wavelength is changed (variable K-vector amplitude). This second approach meets, more exactly, the concept found in the literature as TDM, HT, or TPM [57,58,59]. Diffracted waves can be collected and reconstructed from the holograms at various incidences. A simple way to reconstruct the scattered wave is based on holography: the phase and amplitude of the diffracted wave is directly reconstructed from the hologram and is used to compute the scattering potential at every point of the specimen, according to Wolf (1969). TDM can be performed by applying two different techniques for varying the angle of incidence of light waves illuminating the sample. On one hand, the specimen is rotated by 2π around the optical axis, while on the other hand, the incidence angle is varied by laterally scanning (perpendicular to the optical axis) the illumination point in the back focal plane of the condenser. In 2006, we described the first approach where the specimen has been rotated: a pollen grain [60] and an amoeba. Further works [61,62] have demonstrated the feasibility of the approach based on the rotation of the incident beam. Tomographic phase microscopy of cells, with fully coherent illumination (C-ODT), has been published by Y. Cotte et al. [4] in 2013. Since, several papers have been published which describe an ODT using Partially Coherent Illumination (PC-ODT) [63]. Similarly, another approach using Partially Coherent Illumination, so called Wolf Phase Tomography (WPT) [64], has been proposed. Despite several positive opinions recently expressed about partial coherence tomography, Abbe limitations are still valid.

### 3.2. Future Challenges

A way to overcome the problem of the limitation of resolution, imposed by Abbe law, may be offered by coherent illumination diffraction tomography: C-ODT. A major advantage of coherent image formation is to provide a robust way of deconvolving microscope images. In 2010, Cotte et al. [65] demonstrated that image resolution could be improved beyond the Rayleigh limit by deconvolution of the complex field. An improvement by a factor greater than 1.6 was claimed. This factor can certainly be improved further by the detailed study of the Complex Optical Transfer Function (COTF), comprising both Amplitude and Phase Transfer Function (AOTF and POTF) of the microscopic objective collecting the diffracted wave. A second significative improvement brought by C-ODT is the extension of the accessible domain in K-space for the scattered data: the combination of the K-vector of the irradiating beam and the K-vector of the scattered beam provides a precise determination of the scattering potential, of Fourier terms, in a domain approaching 2Π [4]. Furthermore, part of the problem posed by the missing cone can be solved by a proper combination of the irradiating and scattered light with high NA condenser and MO.

### 3.3. Holography Applied to Microscopy in Biology and Medicine

**Figure 3:**
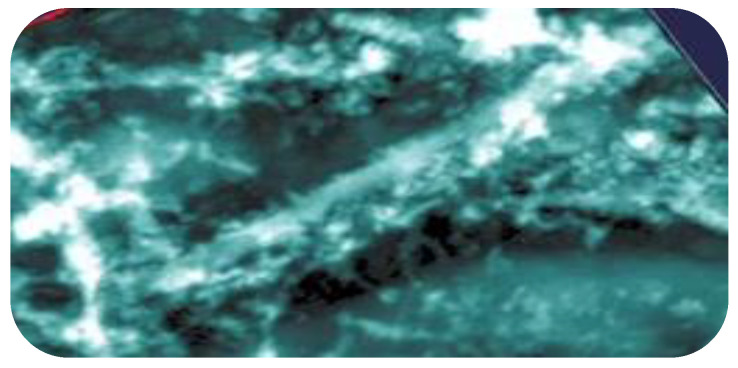
Phase image by TDM of an interpenetrated bundle of neuron dendrites.

**Figure 4:**
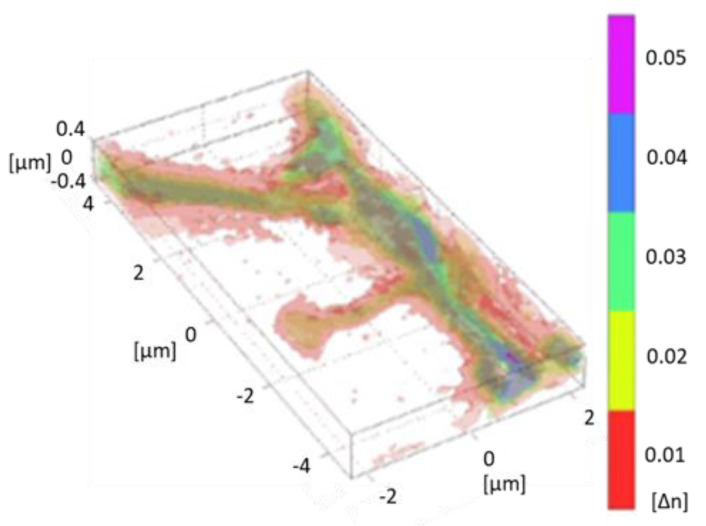
Image of a dendritic spine. Spatial resolution <100 nm [4].

It has been shown that TPM provides super-resolved RI images of cells and tissues. Examples are given in Figure 3. Details of a dendritic spine are shown in Figure 4. The quantitative determination of cell biophysical parameters has opened new paths in biology and medicine: RI variations, combined with the variation of volumes and morphology, have allowed a precise characterization of cell phenotypes. Further details of holography applied to microscopy in biology and medicine can be found in [66,67]

This section was prepared by Christian Depeursinge and Pierre J. Magistretti.

## 4. Quantitative Phase Imaging by Self-Reference on-Axis Holography

### 4.1. Status

QPI is the term given to a cluster of methods that quantify the phase shift that occurs when light waves pass through a more optically dense or thicker object than its background. [1,68]. This powerful tool enables 3D non-destructive imaging, with nanoscale sensitivity, and label-free reconstruction of the morphology of phase objects that are otherwise invisible. Recording the phase information is usually done using interference between a wave that interacts with the sample and a reference wave that does not carry any sample information [69,70]. In this section, we describe the combination between the concept of self-reference on-axis holography and QPI. Extraction of the phase map of the sample is done digitally by one of two approaches; superposition of multiple phase-shifted holograms for the acquisition of the phase [71,72] or using the qualitative description of the phase, captured in a single hologram, for iterative phase retrieval algorithm [73]. The two approaches can be implemented on the same optical apparatus, and the better-suited working mode can be chosen according to the desired imaging requirements.

### 4.2. Current and Future Challenges

**Figure 5:**
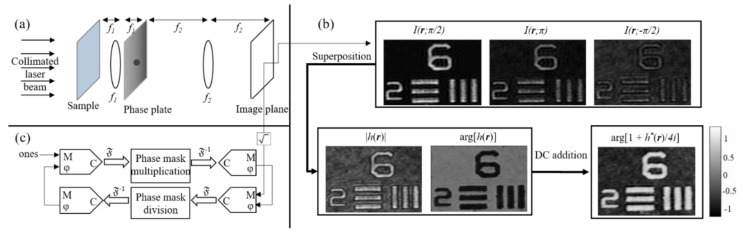
(**a**) Schematic of the self-reference on-axis apparatus used for QPI. (**b**) Flowchart of the phase-shifting process (Adapted from [71]). (**c**) Iterative QPI approach described in the text (Adapted from [73]).

In order to implement a self-reference on-axis holographic device, operating with a coherent light source for accurate phase imaging, we use an experimental configuration of a telecentric spatial filtering system [Figure 5a]. A controllable phase-pinhole plate is positioned at the spectrum plane between the two lenses. Assuming a phase-only object is positioned at the input plane of the imaging system, the intensity captured by the image sensor is,

*I*(*r; ξ*) = ∣*ℱ^−^*^1^{ *ℱ* {*exp*[*iφ*(*r*/*M_T_*)]}(1−*δ*(*ρ*)[1−*exp*(*iξ*)])}∣^2^
≅∣*exp*(*iξ*)+*Σ*_*n*_
*a_n_φ^n^*(*r/M_T_*)∣^2^, (2)

where *ℱ* denotes a two-dimensional Fourier transform, *φ* is the object phase, *ξ* is the pinhole phase, *r* and *ρ* are the object and the spectrum coordinates, respectively, and *M_T_* is the system’s lateral magnification. In order to recover the phase of the examined object accurately, the series in Equation (2) is extracted by capturing three [71] (or two [72]) different phase-shifted images. These images are superposed, and the examined object’s phase is obtained by adding the bias term, as shown in Figure 5b.

Alternatively, Equation (2) can be used to retrieve the phase of the sample by an iterative algorithm that is initialized by a phase-contrast image of the examined object obtained for *ξ = π/2*, as shown in Figure 5c [73]. Clearly, the iterative approach is more attractive due to its rapid acquisition based on a single capture. However, this method is found to be limited to a phase distribution below 2π, and it is also less accurate than the multiple-acquisition approach [71,72].

### 4.3. Advances in Science and Technology to Meet Challenges

**Figure 6:**
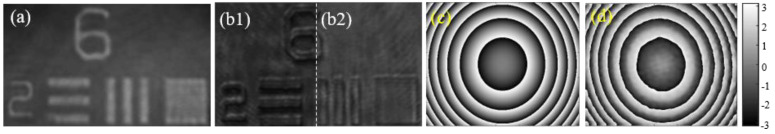
(**a**,**b2**) Reconstructions of thin (OT < λ) phase-only object by the iterative approach when initialized with regular intensity measurement (**b1**) and phase-contrast measurement (not shown), respectively (Adapted from [73]). (**c**) Theoretical and (**d**) reconstructed phase of a refractive lens (OT > λ) by using the phase-shifting method (Adapted from [71]).

Figure 6a,b demonstrate that initializing the modified phase retrieval algorithm with a phase-contrast measurement enables the QPI. An additional advantage of the iterative approach is the ability to record sufficient data for complete recovery of the object’s phase distribution at a temporal rate of the sensor. However, this method cannot work properly for objects having optical thickness (OT) larger than the illumination wavelength. The triple and double-shot method, on the contrary, do not suffer from this limitation as the phase of the sample is measured more than once. Thus, the phase of optically thick objects can be accurately recovered [Figure 6c,d].

To assess the performance of the two QPI modalities, we measured the phase of binary phase-only resolution targets of varying thicknesses from 50 to 300 nm. Table 1 summarizes the estimated thicknesses of the targets, based on 100 measured phase maps for each thickness. From this comparison, it is clear that the multiple-shot approach is more accurate and has a lower degree of uncertainty compared with the iterative, single-shot method. The two QPI modalities presented here can be integrated within a single optical apparatus to achieve a robust phase microscope for a broad range of imaging tasks. Diffraction-limited resolution, fast acquisition, reduced ambient noise, and a full field of view can be easily achieved by the use of the appropriate framework.

### 4.4. Concluding Remarks

Adapting a self-reference scheme into coherent holography for QPI tasks is shown to be practical. In addition to the improved accuracy, compared with conventional interferometry [71], the system’s dual operation provides the basis for future studies based on the two different approaches. Holographic phase recovery and iterative phase retrieval can now be merged in a single optical apparatus.

This section was prepared by Nathaniel Hai and Joseph Rosen.

## 5. Tracking Ultra-Fast Material Transformation with Permittivity Transient

### 5.1. Status

A material’s optical response is defined by its permittivity or the square of the complex refractive index $\sqrt{\varepsilon }=n+ik$. If the evolution of *n* and *k* is experimentally determined with high temporal (an optical cycle *λ*/*c*) and spatial (diffraction limit ~*λ*/2) resolution, the optical response of the material is known and can be predicted. This would help to reveal details of phase transitions [74,75] (solid-liquid, solid-solid), critical volumes for a new phase or void formation, and to unravel one of the unsolved problems in physics: the glass transition and formation of glasses [75] (including metallic glasses and even mono-atomic glasses [76]). In the case of phase transitions triggered by absorption of ultra-short sub-1 ps laser pulses, different hypotheses exist and must be experimentally validated. The absorbed energy, inside a volume with cross-sections of the skin depth (*l_skin_* = 1/*α*, where absorption coefficient *α* = 4π*k*/*λ*) at the peak intensity inside the focal volume is, by definition, *energy/volume = pressure*. Once pressure is larger than the bulk modulus of the surrounding host, a void can be opened [74]. It generates high pressure forms of materials in the surrounding volume, helped by ultra-fast thermal quenching [75]. It is also argued that similar structural modifications can be induced by cavitation in the molten phase [77]. High time/space resolution is required to reveal details of the formation of new materials and their phases using optical pump-probe experiments. To determine a pair of unknowns (*n*,*k*), two independent and simultaneous measurements (probe) in the same volume are required. The Fresnel reflectance *R_s,p_* for the *s*-/*p*-polarizations and *θ* angle of incidence are given as [78]:

(3) $$R_{s}\left(\theta \right)=\left[{\left\{a\left(\theta \right)-cos\left(\theta \right)\right\}}^{2}+b{\left(\theta \right)}^{2}\right]/\left[{\left\{a\left(\theta \right)+cos\left(\theta \right)\right\}}^{2}+b{\left(\theta \right)}^{2}\right]\;$$

(4) $$R_{p}\left(\theta \right)=R_{s}\left(\theta \right)\left\{\left[{\left\{a\left(\theta \right)-sin\left(\theta \right)tan\left(\theta \right)\right\}}^{2}+b{\left(\theta \right)}^{2}\right]/\left[{\left\{a\left(\theta \right)+sin\left(\theta \right)tan\left(\theta \right)\right\}}^{2}+b{\left(\theta \right)}^{2}\right]\right\}\;$$

where $a\left(\theta \right)=1/2\left[\sqrt{{\left\{n^{2}-k^{2}-sin{\left(\theta \right)}^{2}\right\}}^{2}+4n^{2}k^{2}}+\left\{n^{2}-k^{2}-sin{\left(\theta \right)}^{2}\right\}\right]$ and $b\left(\theta \right)=1/2\left[\sqrt{{\left\{n^{2}-k^{2}-sin{\left(\theta \right)}^{2}\right\}}^{2}+4n^{2}k^{2}}-\left\{n^{2}-k^{2}-sin{\left(\theta \right)}^{2}\right\}\right]$. By measuring *R* at two angles *θ*, it is possible to calculate (*n*,*k*). Since energy conservation for reflectance *R*, transmittance *T*, and absorbance *A* are linked by *A* + *R* + *T* = 1, the transmittance can also be used to determine (*n*,*k*).

### 5.2. Current and Future Challenges

**Figure 7:**
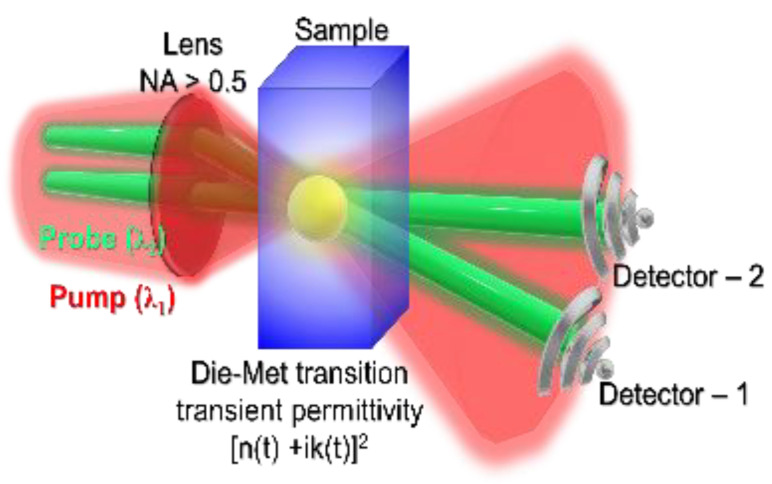
The pump (*λ*_1_) two-probes (*λ*_2_) method allows the recovery of permittivity transient, separation of the phase delay, and absorbance contributions at the wavelength of probe (*λ*_2_). Light-matter interaction from the focal region is fully defined by the instantaneous permittivity (square of the complex refractive index [*n*(*t*) + *ik*(*t*)]^2^). The configuration can be applied to both transmission and reflection modes.

Challenges are mounting to determine (*n*,*k*) from small focal regions and to resolve their fast changes in time. Holographic imaging of the light-matter interaction region is used for the amplitude and phase determination from transmission along pump-probe propagation [79,80,81] or by probing perpendicular to the pump beam [82]. In both cases, the exact determination of (*n*,*k*) is not possible due to one probe beam. In the case of high intensity TW/cm^2^ − PW/cm^2^ ultra-short sub-1 ps pulses, material is transferred into a plasma state fast, within a few optical cycles. Dielectric breakdown is defined when the Re(*ε*) = 0, i.e., *n*^2^ − *k*^2^ = 0 or *n* = *k*. The evolution of *n* and *k* is most dramatic for dielectrics/crystals, which are transferred from a solid state to metallic plasma, hence, a transient state of the Die-Met matter is created and is defined by the instantaneous *ε* [83]. Space-time resolves determination of permittivity, hence (*n*,*k*), empowers experimentalists to follow phase transitions by probing with a light pulse. Due to the fully deterministic nature of femtosecond pulsed laser induced single pulse breakdown/ablation, it would become possible to induce the Die-Met state of matter for all optical control of phase transitions and optical control of the probe beam/pulse. Such new transient metamaterials can extend the application potential of engineered metamaterials based on metal-insulator-metal (MIM) sub-wavelength structures/patterns. For example, an optically induced metal-to-isolator transition (MIT) in VO_2_ is used for optical tunability of metamaterials [84]. Due to the small size/scale of metamaterials, changes to optical properties can be fast, even when driven by local temperature changes/relaxation [85]. Optically induced Die-Met state at the focus (by pump) creates an area/volume where refractive index *n* decreases and becomes smaller than that sample’s *n*_0_, as shown in Figure 7. Consequently, a probe reflected from the air-dielectric interface will not experience a π-phase shift as for the case of light propagation from low-to-high *n* medium. Such large π-phase shifts can be used to characterize phase transitions by reflected probe beams from surfaces and inside volumes where Die-Met transition is induced. The optical method used to observe the phase transition and ionization events often involve bulky interferometry and is based on determination of a cumulative phase change (seen by probe) $\Delta \varphi =\frac{\omega }{c}\int^{\;}\left(n-n_{0}\right)dx,$ where integration is made through the pump-modified region in the x-direction [86]. Generation of free electrons reduces the refractive index, e.g., index of air is defined by electron density *n*_e_ as $n\left(I\right)=\sqrt{1-n_{e}\left(I\right)/n_{cr}}$, where *n_cr_* is the critical plasma density and *I* is the instantaneous intensity of the laser pulse.

Since *n* is related to the phase, the orientation changes of *n* can be expressed as Δ*n*, which is the birefringence. One can envisage that 3D spatial analysis of birefringence related to the orientation distribution of mass density, material porosity, composition, etc., can be made possible from measurement of phase changes. Such 3D tomography of refractive index, and its local changes on micro- and nano-scales, is a sought after technique. The orientation dependence of birefringence Δ*n* can be, in principle, separated from that of dichroism Δ*α*, which is due to anisotropy of absorbance, as recently demonstrated [87]. Simultaneous acquisition of images at four linear polarizations is what is required for detection of anisotropy in refractive index or/and absorption. Recently, such cameras have become commercially available for visible spectral range and are expected to advance microscopy and aerial imaging, including hyperspectral imaging.

### 5.3. Advances in Science and Technology to Meet Challenges

New imaging techniques based on multidimensional characterization of events changing in 3D volume, time, and spectrum are emerging and can solve previously encountered obstacles of characterization of micro-volumes via pump-probe interferometric techniques [82,86]. With the recent developments in imaging technologies to observe fast transient events in five dimensions [88] and rapidly converging (<5 iterations) phase retrieval approaches [89] using a single camera shot, we believe that it will be possible to obtain additional information about the rapid events occurring in a small volume in space, without the use of bulky time resolved interferometry.

### 5.4. Concluding Remarks

Observation and understanding of phase transition events, during light-matter interactions in confined volume, is important for the development of advanced lithography tools. Conventional interferometry has been widely used for quantifying the above events, which we believe are limited by the information bandwidth and are bulky and difficult to implement. In the coming years, we believe that the current methods will be replaced by single shot, elegant, computational optical methods with higher information bandwidth [88,89].

This section was prepared by Saulius Juodkazis, Soon Hock Ng, and Vijayakumar Anand.

## 6. Three-Dimensional Quantitative Phase Imaging

### 6.1. Status

Various 3D microscopy techniques have been utilized for the study of cells and tissues. Of these, QPI has demonstrated potential as a label-free and high-throughput imaging method [5]. Because 3D QPI utilizes the RI for endogenous image contrast, it does not require labeling procedures and benefits from rapid, long-term assessment of biological specimens in their native states. From the multiple measurements of two-dimensional holograms, a 3D RI tomogram is reconstructed by inversely solving the Helmholtz equation. Two decades after the invention of holography [90], a theoretical framework [17], referred to, in short, as ODT or HT, was established by E. Wolf and then experimentally demonstrated by others [91,92]. Applications of HT in biomedical research expanded in the 2000s. Early HT systems were operated by tilting the incident angles of a plane wave using motorized [59] or galvanometric [4,61] mirrors. Spatial light modulators, such as a liquid-crystal phase modulator, were used in HT [93,94].

### 6.2. Current and Future Challenges

#### 6.2.1. Image Quality

The image quality of 3D QPI is determined by spatial resolution and by RI accuracy and precision. Lateral spatial resolution is primarily determined by the wavelength of a light source and the numerical apertures of both the condenser and objective lens. The axial spatial resolution of 3D QPI is inferior to its lateral resolution because of uncollected side- or backscattering signals. This issue, known as the missing cone problem, generates artifacts in 3D images along the axial direction via the underestimation of RI values. This limited axial resolution becomes important when measuring optically thin samples such as red blood cells [95] and bacteria [96].

RI accuracy and precision are important, particularly when quantitative analysis of cellular or subcellular components is necessary. RI accuracy deteriorates when the RI contrast between a sample and surrounding medium is high, as reconstruction algorithms rely on the weak scattering assumption and RI values that gradually vary in space [97]. When the RI contrast is high, multiple light scattering becomes significant and leads to an underestimation of RI values. RI precision is determined by the level of speckle noise, the ability to precisely control the angle of an illumination beam without wavefront distortions, the good optical transfer function of an imaging system, and the performance of an image sensor.

#### 6.2.2. Imaging Throughput and Data Size

Although the imaging speed of 3D QPI is sufficiently high for capturing live cell dynamics, its imaging throughput requires improvements. A field of view is approximately limited to 100 × 100 µm^2^. This is because of the limited space-bandwidth product of QPI [18] and the considerable computation necessary during the reconstruction process. To image a larger area, tiled images can be measured and stitched [98]. However, sequential imaging and stitching are time-consuming, and handling large-sized image data consumes considerable computational power.

#### 6.2.3. Interpreting 3D Data

Although 3D RI images readily provide structural information for visualizing subcellular components, the lack of molecular specificity in RI complicates straightforward interpretation in biology. In many studies, both RI maps and fluorescence images have been acquired and used together for correlative analysis [99,100]. However, this requires using exogenous labeling agents, which diminishes the benefits of using QPI.

Given that RI value is linearly proportional to a protein concentration [101], 3D QPI has been used for quantitative analysis of cellular dry mass. However, this feature has not been fully exploited because of the difficulties in segmenting cellular membranes or subcellular organelles, as they share ranges of RI values and are not readily distinguishable.

### 6.3. Advances in Science and Technology to Meet Challenges

Recent interdisciplinary advances have enabled these challenges to be addressed. These include theoretical advances, new optical instrumentation, and significant improvements in computational methods. To enhance image quality, several studies have developed efficient reconstruction algorithms to consider multiple light scattering. These include beam propagation [102], multi-slice Born approximation [103], and gradient computations based on the Lippmann–Schwinger equation [104]. Imaging throughput has been improved by reducing the loss of spatial bandwidth [105,106] and by utilizing Fourier ptychography with LED arrays [107,108]. Advances in optical systems have also contributed to 3D QPI. The introduction of a digital micromirror device to 3D QPI enables stable but rapid control of the illumination beam [109,110], simple setups [106,108,111].

**Figure 8:**
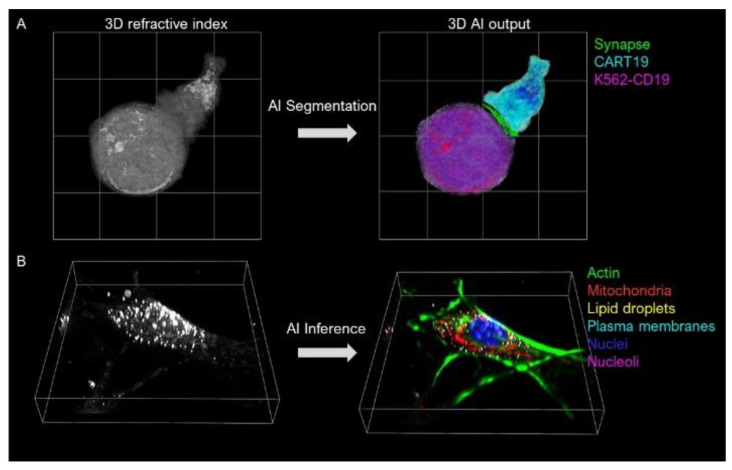
AI-based analysis of 3D QPI. (**A**) Segmentation of immunological synapse formation. (**B**) Retrieving molecular information from unlabeled cells.

Revolutionary advances in AI have addressed several challenges of 3D QPI. Deep learning approaches have been used in image acquisition and reconstruction, including denoising and deblurring [112]. Reconstructed images have also been analyzed, with the aid of AI, for biological studies, including cell type classifications (Figure 8a) [113] and segmentation [114]. Recently, obtaining molecular information from unlabeled live cells has been realized by training a network architecture with RI and fluorescence image pairs in order to extract molecular information from unlabeled RI tomogram data (Figure 8b) [115].

### 6.4. Concluding Remarks

During the last decade, 3D QPI research has focused on developments in optical methodology and reconstruction algorithms. We are currently witnessing the transition of 3D QPI techniques from optical tables to wet benches in biological labs and the use of these techniques as critical imaging methods for various applications. For example, cytotoxicity assays [116], lipid quantification [117], and phase separations [118] are active and rapidly growing fields of interest.

Multidisciplinary attempts have resolved many technical challenges and have enabled 3D QPI to be more accessible to researchers. However, several challenges remain before 3D QPI can be fully exploited for use in biomedicine. The technique awaits more interesting developments. For example, simultaneous optical imaging and manipulation of live cells would open new research directions [119,120]. Using genetically expressed RI contrast agents [121] may also be exciting. Its expanding cooperation with various research areas will make 3D QPI a complementary imaging tool for regular biological studies.

This section was prepared by Moosung Kee and YongKeun Park.

## 7. 3D QPI: Integrated Dual-Mode Tomography

### 7.1. Status

The methodological practice of DHM is utilized to obtain two-dimensional (2D) phase profiles, which are then mapped onto the 3D Ewald’s sphere; this is then extended to 3D QPI by adapting the back projections principle, which is similar to the computational model practiced in a conventional x-ray-computed tomography technique [122,123]. This holography-based 3D QPI method is referred to by various terminologies as discussed in the introduction. The HT technique has the potential ability to reconstruct the quantitative (3D) RI distribution of biological specimens in their native conditions. The RI values are a crucial parameter to determine biophysical properties and cell metabolic changes; these are directly correlated with dry mass, wet mass, protein concentration, elasticity, cell division, infection, etc. [30,60,124]. The HT technique has another attraction for biological researchers because of its label-free measurement approach.

### 7.2. Current and Future Challenges

In general, the HT technique can be implemented either by adapting the synthetic aperture-based beam rotation (BR) approach or by the sample rotation (SR) approach [46]. The spatial frequencies imposed by the finite numerical aperture of the objective lens are collected, and combining the acquired frequency bands corresponding to different angles to extend the spatial frequency coverage. In the BR approach, a conventional galvanomirror device was adapted to control the illumination beam; later alternative devices such as digital micromirror devices (DMDs) and spatial light modulators (SLM) have evolved for use as the beam steering device in the HT system [101,110,125]. In the BR approach, one can improve the lateral spatial resolution of the imaging system, and in this technique, the spatial frequency collection constraint is commonly referred to as the missing cone problem [7]. Another possibility to achieve HT is by the SR approach; under the static illumination beam, the axial resolution can be improved by capturing a series of sample’s information at different rotation angles by rotating the sample [122]. In the SR approach, microcapillary or micropipette are some such tools controlled mechanically for the sample rotation. The usage of mechanical rotation tools limits the sample rotation angle and results in the missing frequency coverage. The limited missing frequency constraint in SR approach is referred to as the missing apple core (MAC) problem [126]; the mechanical rotation makes the system susceptible to aberrations [127]. In the SR approach, the MAC problem can be overcome by achieving full-angle (360°) sample rotation, and this technique has been demonstrated by adapting holographic optical tweezers [128] as a potential tool for the sample rotation [129]; consequently, an isotropic frequency coverage is achieved. Several computational methods have been demonstrated to enhance the missing core data problem in each of the approaches [7]. However, there are still limitations in experimenting on the combined architecture to get the advantages from both BR and SR approaches, and this is due to the limitations imposed by each of the techniques.

### 7.3. Advances in Science and Technology to Meet Challenges

**Figure 9:**
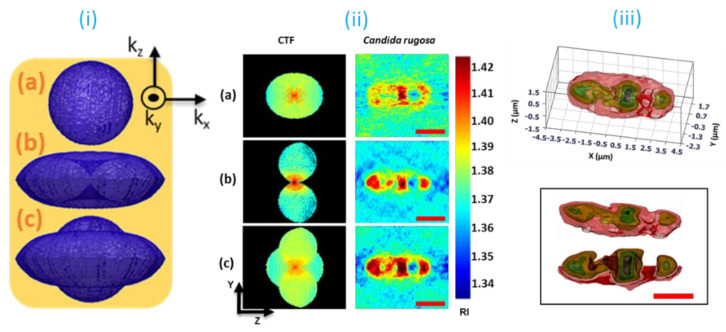
(**i**): Comparison of coherent transfer functions (CTF) of (**a**) BR, (**b**) SR, and (**c**) IDT. (**ii**): Comparison of slices of 3D refractive index distribution of *candida rugosa* and the experimentally obtained CTFs corresponds to (**a**) SR approach, (**b**) BR approach, and (**c**) IDT approach. (**iii**): 3D illustration of *candida rugosa* at sub-cellular structural views; the different colors represent the different organelles of the cell. Scale bars: 2 µm. Adapted from [130].

It is important to have an integrated dual-mode tomography system, capable of collecting the spatial frequencies of both the BR and SR approaches in a single system which is much needed to overcome the weaknesses of both of these approaches. An attempt has been made to combine BR and SR approaches with mechanically controlled sample architecture. However, this implementation is not feasible for the biosamples because of several limitations imposed by the experimental architecture. Later, in the technical advancements, an integrated dual-mode tomography (IDT) system is developed by combining holographic optical tweezers (HOT) [57,58,59] with synthetic aperture-based DHM. The IDT system that has been developed is capable of collecting and combining the spatial frequencies of full-angle sample rotation, shown in Figure 9i(a), with those of BR approach, shown in Figure 9i(b). Consequently, an enlarged spatial frequency coverage is achieved, as shown in Figure 9i(c), and has resulted in high-quality image reconstruction. The IDT technique is validated using the live *candida rugosa* (commonly called yeast) as a testing sample, and the obtained results, corresponding to SR, BR, and IDT approaches, are compared in Figure 9ii. The sub-cellular structural views are illustrated in Figure 9iii.

### 7.4. Concluding Remarks

This integrated dual-mode tomography approach is highly useful to analyze a single live cell with higher resolution at its native cell culture environment. The IDT system offered the extended spatial frequency coverage by collecting and combining the spatial frequencies of the full-angle sample rotation method, along with those of the beam rotation method for free-floating live cell imaging and analysis. Consequently, the IDT imaging system can reconstruct the 3D RI distribution with an accuracy of 0.003, and a novel unidentified flying object (UFO), such as shaped coherent transfer function, was obtained. The experimental resolution was estimated, and the axial resolution and lateral resolution were approximated as 310 nm and 150 nm, respectively [130]. The IDT imaging system does not require any complicated image-processing procedures for the 3D image reconstruction. It is strongly believed that the IDT technique has prospective applications in the biomedical field, where single or multiple live cell analysis is much needed, for the analysis, as well as noninvasive biological studies. Different shaped samples are also possible to study in the IDT imaging system by adapting a suitable trapping technique [120]. The IDT system has more flexibility in controlling the free-floating sample, and it is possible to extend the spatial frequency coverage along the axial direction. The continuous development and progressive solutions by the researchers in computational, experimental, and artificial intelligence algorithms has brought enhanced measurement accuracy to technique, increased certainty of analysis [112,113,114,115,131,132,133], as well as access to new multimodal techniques and functionalities [134,135,136,137,138,139,140].

This section was prepared by Vinoth Balasubramani and Chau-Jern Cheng.

## 8. Ultra-Scale High-Contrast Holographic Tomography

### 8.1. Status

Originally, phase imaging was developed to obtain high contrast images of thin 2D biological samples [141]. For these samples, intensity variations are minimal, and imaging the sample structure is only possible through the phase. The most-used form of QPI is based on digital holography [142] using phase stepping or off-axis illumination. Combining quantitative phase measurements from different directions through computed tomography resulted in the extension of QPI to three dimensions, most commonly known as ODT or HT.

As described in Section 7, in HT, diffraction patterns are obtained in two ways: either by the SR or by the BR approach. The SR geometry has the advantage of a simple system design and close to isotropic resolution. However, it has limited compatibility with in-vivo imaging and is fundamentally limited in coverage of the frequency support of the object limiting the maximum attainable resolution. Varying the illumination direction is more amenable for high throughput in-vivo imaging and does capture up to the maximum frequency range. However, the BR configuration has limited resolution in the depth direction, due to the ‘missing cone’ (limited angle of projections) in the frequency support of the object.

**Figure 10:**
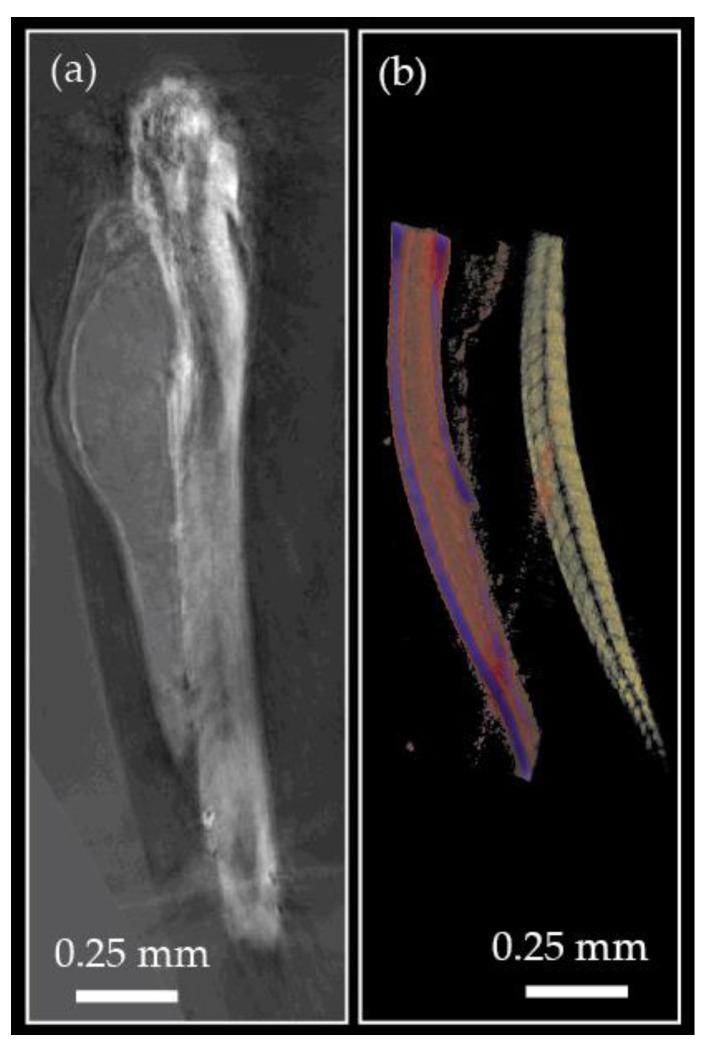
(**a**) Multi-scale HT zebrafish image [143]. (**b**) Refractive index (red) and polarization contrast (yellow) HT zebrafish tail image [149].

Initially, HT was applied for high resolution imaging of cells and small multicellular organisms. More recently, HT has been applied to millimeter-sized transparent materials, such as lenses [143]. Using optical tissue clearing, HT has become possible with larger pieces of tissue, such as cleared zebrafish [144], as shown in Figure 10a. In all these applications, HT has shown its unique capability for obtaining high resolution images in 3D.

### 8.2. Current and Future Challenges

#### 8.2.1. Ultra-Scale HT

One of the challenges of HT is to obtain high resolution images over a large field of view, i.e., multi-scale imaging. In HT, this is achieved by recording holograms using objective lenses with low magnification and high NA. To achieve hardware-limited performance, imaging has to be performed close to the space-bandwidth limit of the detector. For off-axis holography, this can be achieved by eliminating the zero order, autocorrelation, and conjugate image [145]. Further progress in multi-scale HT is aimed at resolution enhancement through filling the frequency support of the object more efficiently, e.g., by combining sample rotation and illumination scanning [146,147] or counteracting the missing cone artifact [148].

Although multi-scale HT is useful for obtaining high quality 3D images, it suffers from long acquisition times and does not allow for in-vivo or dynamic imaging. To address this challenge the goal is to develop ultra-scale HT, i.e., HT with optimized throughput (defined as the ratio of volume per second over resolution). The realization of ultra-scale HT would allow the application of HT to entirely new areas, such as in-vivo whole organism imaging at cellular resolution and large scale tissue histology.

The realization of ultra-scale HT requires improved system design that allows for rapid control over illumination direction and the use of (multiple) high speed cameras to rapidly cover the entire frequency support. Efforts in this direction have been made, but they are currently limited in speed and angular coverage [109]. Similar to multi-scale HT, ultra-scale HT requires that the available bandwidth, fundamentally limited by the hardware, is used as efficiently as possible. Further enhancement of the throughput can only be achieved by estimating frequencies beyond the available bandwidth, e.g., using super-resolution techniques.

#### 8.2.2. High contrast HT

The challenge with HT as a label-free technique is to obtain sufficient contrast in images of low contrast tissues. Since, for large scale HT optical clearing, it is of paramount importance to remove absorption, scattering, and tissue refraction, this also leads to lowering of the intrinsic phase contrast and, hence, to a low dynamic range.

One way to improve contrast in HT has focused on optimizing the signal to noise ratio (SNR) of the intrinsic phase signal, e.g., through the use of more optical power [150]. However, this requires cameras that can measure high optical powers with high linearity. Another way is through reducing the effects of noise by using low dark-count cameras or reducing speckle noise by off-axis sample placement, combined with digital refocusing [151]. Yet another tactic is to create contrast by imaging the intensity modulation caused by phase differences between polarization states. This polarization contrast HT has shown high contrast imaging of birefringent muscle structures in zebrafish [151] and imaging of optical retardation and birefringent tensor elements [152].

Another way of improving HT contrast is by utilizing the amplitude information of the scattered field. Potentially, this could yield information about the 3D distribution of endogenous or exogenous (labeled) absorbing markers. In the context of polarization contrast HT the amplitude information can be used for imaging diattenuation, which has already been shown to yield information about the brain tissue structure [153], as shown in Figure 10b.

Future developments in contrast enhanced HT are to exploit the time varying refractive index fluctuations for the assessment of the dynamic behavior from the level of within the cell to the entire small organism [154]. However, enabling temporal tissue contrast requires the realization of ultra-scale HT. Similarly, measurement of the dynamic tissue response to mechanical deformation can yield improved contrast. Similar to other elastography modalities (e.g., ultrasound or optical coherence tomography), this could provide spatially resolved mappings of the stiffness and/or the dynamic load response of tissue.

### 8.3. Advances in Science and Technology to Meet Challenges

The challenge to achieve ultra-scale and high-contrast HT can be met through advances in system hardware and computational tools.

Given the good availability of high power coherent sources, there is a clear demand for large well depth cameras to enhance the SNR [150] and make high-contrast in-vivo imaging possible. On the other hand, advances in acquisition speed improvement need to be matched by rapid illumination direction modulation.

Conventional phase-sensitive HT reconstruction algorithms have to be improved to remove image artifacts caused by tissue refraction, optical aberrations, and multiple scattering. This optical full-wavefield inversion would create higher resolution images and thereby boost the effective image throughput.

To create improved contrast, multiple physical properties (absorption, phase, birefringence) need to be incorporated in the reconstruction algorithm by developing multi-physics tomographic reconstruction algorithms.

### 8.4. Concluding Remarks

QPI methods have the unique advantages of high resolution label-free 3D imaging. The challenge is to push the boundaries, in image contrast and throughput, to open up new application areas, such as live whole organism imaging and large volume cleared tissue imaging, and make visible what has not been seen before.

This section was prepared by Jeroen Kalkman.

## 9. Metrology Aspects in 3D QPI

### 9.1. Status

The key advantage of the 3D QPI is its capability to retrieve, non-destructively and label-free, 3D distribution of RI in transparent and weakly scattering samples. This quantity is relevant in many applications, but most notably, RI distribution is currently of high interest in biomedicine [5,7,58], as it is related to the dry mass density distribution, which provides insight in the cells’ and tissues’ morphology and their changes. The 3D QPI is explored with a variety of methods, with the limited-angle ODT leading the way in terms of accuracy of the reconstructions and commercialization efforts in the area of biomedicine. With the rise in popularity of the 3D QPI techniques, including multiscale and multimodal approaches [98,151,152], as well as the use of machine learning to facilitate both reconstruction process and data analysis [67,155,156], it is of utmost importance to measure and consider the uncertainty of the underlying data. It is clear that cross-referencing the results between different techniques, optomechatronic systems, and laboratories, as well as the future databases, has immense value and can only be achieved with proper metrology.

Major sources of errors in 3D QPI can usually be attributed to projection acquisition (sampling, sensitivity, and noise), system parameters (transformations between sample, projection, and reconstruction space) and reconstruction process (method, approximations). In reality, quality assessment based on these error components is impractical due to their complex interplay and contribution to the measurement result.

In more mature industrial and medical 3D imaging techniques even dimensional traceability of the measurements is an active area of research [157,158]. Recent advancements in 3D printing have enabled a range of geometrical and anthropomorphic phantoms focused on recreating challenging aspects of real volumetric experiments [159], yet there is no universal solution for metrology in 3D QPI.

### 9.2. Current and Future Challenges

**Figure 11:**
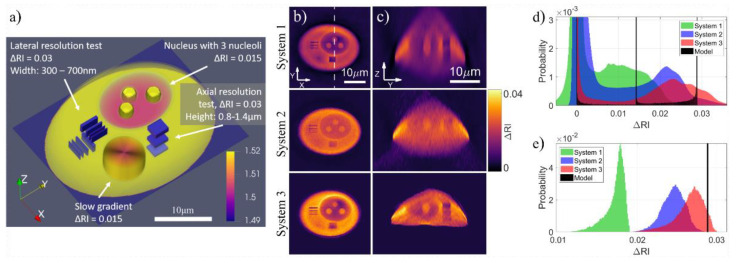
(**a**) CAD model of the 3D cell phantom. (**b**,**c**) Cross-sections of the 3D RI distribution of the phantom measured using three different holographic tomographs with a limited angle of projections. (**d**,**e**) Histograms of the ΔRI in case of full measurement volume (**d**) and manually segmented single nucleoli (**e**).

Modern QPI instruments often have proprietary hardware and software specifically designed to enhance measurement fidelity—a reality that argues in favor of end-to-end testing, especially considering that the reconstruction process is not quite robust, and error varies greatly depending on the features of interest. This problem is well illustrated in Figure 11, where the 3D cell phantom is investigated with three different holographic tomographs and, both local and global, histograms of ΔRI (and thus inferred dry mass) and reveals significant differences in the measurement results [160,161,162].

Further developments in standardized specimens—be it cells, cell clusters, tissues, or technical phase objects, as well as quality metrics for determining the metrological characteristics of a given instrument, are required in order to incorporate this metrological knowledge to improve the hardware, software, analysis, and what is most important: dissemination and providing reliable 3D QPI databases for intercomparison between laboratories and remote diagnostics (e.g., digital phase histopathology).

Currently, the main challenge on the way to adequate metrological assessment of 3D QPI systems is access to validated phantoms. The manufacturing requirements put forward by the high resolution and sensitivity to the refractive index are quite demanding (usually in the range of 100 nm and 10^−4^, respectively), especially in a form-factor suitable for systems devoted to biological specimen. Moreover, the resulting structure has to be validated, and while some aspects of characterization can be readily available (e.g., electron microscopy or profilometry for geometry analysis), there is no single technique that could provide 3D morphology and corresponding refractive index values of required fidelity to serve as reference. This significantly hinders development and commercialization of truly quantitative 3D QPI systems.

### 9.3. Advances in Science and Technology to meet Challenges

Suitable test structures can be obtained thanks to the advances in micro- and nano-scale fabrication in the area of 3D printing. In particular, the two-photon polymerization method seems to fulfill the requirements set by the 3D QPI metrology [163]. It enables fabrication of complex structures with sub-µm resolution and locally adjustable refractive index (based on the degree of conversion of the monomer) in the scope suitable for 3D QPI. It is also capable of producing multiscale, multimodal structures with adjustable scattering, fluorescence, or birefringence by exploiting new or doped materials in order to faithfully recreate cell clusters, tissue slices, or even small animals [164].

When the phantoms become widely available, the next challenge is related to the reporting of the metrological data in a complete, yet concise manner. A promising approach is to generalize the concept of the 3D optical transfer function [165,166,167] to the 3D instrumental transfer function as in surface metrology [168]. Such quantification of the 3D frequency response of the system to the particular volumetric sample and with respect to its modalities should account for all imperfections during the measurement and reconstruction process.

### 9.4. Concluding Remarks

A wide range of currently used and newly developed 3D QPI methods with the ever-expanding functionalities will benefit from the proper determination of accuracy and precision of instruments and uncertainty of the measurements. As current fabrication methods exceed the 3D QPI capabilities, challenges in metrology are shifting towards validation and standardization of the phantoms and establishing metrological guidelines. If done right, it will open up a new chapter in imaging, measurement, and diagnostics, thanks to the cross-referencing a range of physical properties between QPI techniques and beyond.

This section was prepared by Malgorzata Kujawinska, Michal Ziemczonok and Arkadiusz Kus.

## 10. From Computational to Neural Microscopy

### 10.1. Status

**Figure 12:**
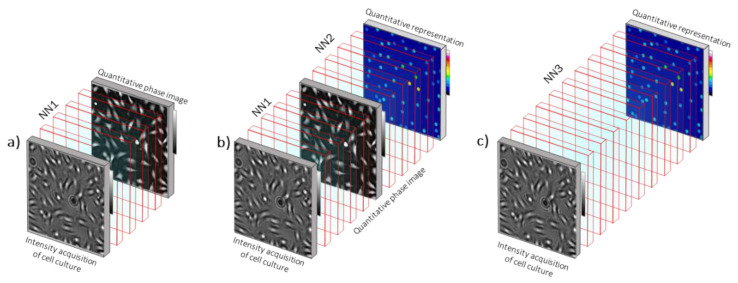
Neural microscopy framework for quantitative phase imaging. (**a**) NN1 performs reconstruction of quantitative phase image from intensity acquisition. Here, as an example, the sample is a culture of adherent cells. (**b**) NN2 infers a quantitative representation from the phase image. Here, as an example, NN2 maps the phase image of adherent cells into an image that encodes, simultaneously, cell positions and dry mass measurements. (**c**) NN3 directly infers the quantitative representation from intensity acquisition. Phase image reconstruction is discarded. Such NN could be all-optical (adapted from [176]). Light is then transmitted, through the sample, towards a fabricated NN that infers an image of the quantitative representation.

Here, we emphasize the ongoing evolutions that transform microscopy into a novel neural microscopy. Recent advances in computational microscopy have led to the preponderant place of algorithms in microscopy experiments. Microscopy can be now considered as a whole, along with the joint design of an optical setup and algorithms [169]. It is now envisioned that deep learning solutions can advantageously replace conventional algorithms. Several convolutional neural networks (NNs) have proven to be very efficient in conducting many tasks, e.g., image processing and inverse problem solver for image reconstruction [170,171]. If neural networks are intended to replace most—if not all—conventional algorithms, it is thus possible to define a novel neural microscopy as being the joint design of optical hardware and neural networks. Looking at the ongoing evolutions of deep learning applied to microscopy, we found three important evolutionary leaps, which can settle the concept of neural microscopy (Figure 12). These are (i) NNs to form an image of the sample, (ii) NNs to infer quantitative or symbolic representation of the sample, and (iii) all-optical diffractive NNs directly analyzing the sample without image acquisition. In the following, we discuss how these evolutions apply to the sub-field QPI [5].

### 10.2. Current and Future Challenges

Reconstruction of phase images from intensity with NNs have been recently reviewed in [172] (Figure 12a). For instance, physics-informed deep learning solutions can run regularization, in place of gradient-descent schemes, to recover the phase image [173]. Interestingly, several papers have found analogy between NN and beam propagation methods [102,174]. The inverse problem can thus be solved using a diffractive NN, where the weights of each layer are the unknown refractive indexes of a slice of the object and the matrix product between each layer are replaced by a formulation of the light propagation. The 2D or 3D refractive distribution of the object can then be retrieved by training the diffractive NN with experimental results [175]. All together, these recent works demonstrate that NNs can encode, together, the object and the physics of light scattering, allowing 2D or 3D phase image formation. Using deep learning frameworks for image formation is advantageous in terms of computation time, since GPU speed-up is straightforward. Note that the use of deep learning is not impaired by generalization issues usually faced by deep learning if data fit is insured [173,175]. However, phase images obtained through NN are rarely compared with that obtained with QPI methods of reference. For NN-based phase image formation to be accepted in the realms of QPI, more metrology studies have to be carefully conducted. Phase images, reconstructed with NNs, can next be transferred to other NNs specialized in image processing. These interconnections of NNs, coupled to an optical setup, thus form a uniform and coherent development framework, which settle the novel neural microscopy.

Another evolutionary leap that further defines the concept of neural microscopy is the development of novel NNs capable of generating quantitative representation. Such NNs will be able to map a phase image into an image that encodes, simultaneously, the object position and the measurements (Figure 12b). The latter can be an image with dots at the position of cells, with the dot gray levels corresponding to the cell dry mass measurement. Such a NN solution offers a fast means to infer quantitative measurements. For faster computation, one could rely on an intermediate image different from the sample image itself (Figure 12c). This moves neural microscopy away from QPI, since imaging would be discarded.

Ultimately, optical setup and computers can be discarded. Ozcan et al. introduced an all-optical diffractive NN able to compute a classification task from spatial information of objects [176]. In line with this development, novel all-optical diffractive NNs could perform quantitative measurements (Figure 12c). Again, this approach, which can be considered within the concept of neural microscopy, will be a move away from conventional QPI.

### 10.3. Advances in Science and Technology to Meet Challenges

A pertinent choice to develop all software elements of the novel neural microscopy is the Python language. The latter is already used to develop acquisition microscopy software and is closely linked to the development of deep learning since the advent of NNs. In the framework of neural microscopy, the use of Python can thus solve a long time problem associated with microscopy, i.e., the lack of a consistent software ecosystem able to conduct acquisition, image reconstruction, and analysis [177].

There is however an important issue for neural microscopy to be applied in the sub-field of QPI. The quantification ability of NNs remains to be studied and validated. This necessitates the development of new methods and characterization means to better support metrology studies involving NNs.

### 10.4. Concluding Remarks

We define a concept of neural microscopy, relying heavily on neural networks with the ability to perceive and analyze at a very fast rate. It is forming a consistent framework for the development of novel phase imaging techniques. However, developments that favor computation speed could move neural microscopy away from QPI as we know it today, particularly if quantification no longer relies on image formation.

This section was prepared by Cédric Allier.

## 11. Conclusions

Quantitative Phase Imaging is a very broad area. The label-free imaging capability of QPI technique enables 2D and 3D imaging with high accuracy retrieval of various physiological parameters of biological cells and their internal organelles, including morphological ones, such as shape and volume, and biophysical parameters, such as RI distribution, dry mass, and more, which can then be analyzed in greater depth. It is also important in material science and industry in which the control of functional distribution of refractive index in microstructures is of high interest. The 3D HT technique has emerged as one of the most powerful 3D QPI methods for the investigation of biological cells and tissues in a non-invasive manner. The HT has just begun to garner appreciation for its competencies, and in the near future, this technique will certainly clench an irreplaceable role in biological studies and analyses. The acceptance of HT in the biomedical community has enlarged, mainly due to the readiness of the commercial holographic tomography systems [178,179,180]. It is also supported by establishing metrological guidelines for QPI systems, which confirms the quantitative character of the results [160,161,162]. At present, 3D QPI has enhanced its implementations by combining techniques, such as fluorescence or Raman imaging, into multimodal operations [135,136,137,138,139,140,141,152,153,155]. Recent developments in artificial intelligence algorithms and machine learning approaches are now the focus in 3D QPI systems, aimed at improving system architecture and measurement accuracy in a more effective way [112,113,114,115,131,132,133,176,177].

This Roadmap article is comprised of 9 sections, contributed by prominent experts in the field, to provide an overview of various aspects of QPI. Although most of the sections are focused on the most popular QPI implementations, through digital holographic microscopes and optical diffraction tomographs, the presented challenges and trends in 2D and 3D QPI development are much more general, and they can be referred to the microscopes based on all phase measurement methods. Each of the section refers to particular aspects of QPI, however it is relatively easy to compile the most important common challenges for 2D and 3D QPI. This includes (i) the demand for increased phase image quality, including spatial lateral and axial resolutions, and high signal-to-noise ratio, (ii) continuously increasing imaging throughput and data size, due to combined high resolution and large size of the sample, as well as multi-modality of measurements, (iii) taking into account the metrological aspects of QPI instrumentation, which allows corroborating measurements across instruments, laboratories, and geographic sites, (iv) improved means for biomedical interpretation of QPI data, and (v) combining QPI with artificial intelligence to create 2D or 3D neural microscopy, relying heavily on neural networks with the ability to perceive and analyze data at a very fast rate.

This section was prepared by Malgorzata Kujawinska and Vinoth Balasubramani.

## Figures and Tables

**Table 1:** Mean measured thickness of a binary phase object using the different QPI methods.

Iterative	Phase-Shifting		Expected Thickness
	2 Exposures	3 Exposures	
45.3 ± 5.6 nm	45.4 ± 3.7 nm	48 ± 6.1 nm	50 nm
119 ± 8.1 nm	99.8 ± 10.5 nm	94.7 ± 11.3 nm	100 nm
211 ± 7.6 nm	200 ± 7.7 nm	199 ± 6.4 nm	200 nm
310 ± 9.5 nm	298 ± 6.3 nm	300 ± 3.3 nm	300 nm

## Data Availability

Not applicable.

## References

[1] Popescu G. (2011). Quantitative Phase Imaging of Cells and Tissues.

[2] Shaked N.T., Zalevsky Z., Satterwhite L.L. (2012). Biomedical Optical Phase Microscopy and Nanoscopy.

[3] Kemper B., Von Bally G. (2008). Digital holographic microscopy for live cell applications and technical inspection. Appl. Opt..

[4] Cotte Y., Toy F., Jourdain P., Pavillon N., Boss D., Magistretti P., Marquet P., Depeursinge C. (2013). Marker-free phase nanoscopy. Nat. Photonics.

[5] Park Y., Depeursinge C., Popescu G. (2018). Quantitative phase imaging in biomedicine. Nat. Photonics.

[6] Sung Y., Choi W., Fang Y.C., Badizadegan K., Dasari R., Feld M. (2009). Optical diffraction tomography for high resolution live cell imaging. Opt. Express.

[7] Balasubramani V., Kuś A., Tu H.Y., Cheng C.J., Baczewska M., Krauze W., Kujawińska M. (2021). Holographic tomography: Techniques and biomedical applications [Invited]. Appl. Opt..

[8] Wang Z., Millet L., Mir M., Ding H., Unarunotai S., Rogers J., Gillette M.U., Popescu G. (2011). Spatial light interference microscopy (SLIM). Opt. Express.

[9] Nguyen T.H., Kandel M.E., Rubessa M., Wheeler M.B., Popescu G. (2017). Gradient light interference microscopy for 3D imaging of unlabeled specimens. Nat. Commun..

[10] Bon P., Maucort G., Wattellier B., Monneret S. (2009). Quadriwave lateral shearing interferometry for quantitative phase microscopy of living cells. Opt. Express.

[11] Lue N., Bewersdorf J., Lessard M.D., Badizadegan K., Dasari R.R., Feld M.S., Popescu G. (2007). Tissue refractometry using Hilbert phase microscopy. Opt. Lett..

[12] Trusiak M., Mico V., Garcia J., Patorski K. (2016). Quantitative phase imaging by single-shot Hilbert–Huang phase microscopy. Opt. Lett..

[13] Zheng G., Horstmeyer R., Yang C. (2013). Wide-field, high-resolution Fourier ptychographic microscopy. Nat. Photonics.

[14] Sun J., Chen Q., Zhang J., Fan Y., Zuo C. (2018). Single-shot quantitative phase microscopy based on color-multiplexed Fourier ptychography. Opt. Lett..

[15] Paganin D., Nugent K.A. (1998). Noninterferometric phase imaging with partially coherent light. Phys. Rev. Lett..

[16] Gabor D. (1949). Microscopy by Reconstructed Wave-Fronts. Proceedings of the Royal Society of London. Ser. A Math. Phys. Sci..

[17] Wolf E. (1969). Three-dimensional structure determination of semi-transparent object from holographic data. Opt. Commun..

[18] Cuche E., Marquet P., Depeursinge C. (1999). Simultaneous amplitude-contrast and quantitative phase-contrast microscopy by numerical reconstruction of Fresnel off-axis holograms. Appl. Opt..

[19] Marquet P., Rappaz B., Magistretti P.J., Cuche E., Emery Y., Colomb T., Depeursinge C. (2005). Digital holographic microscopy: A noninvasive contrast imaging technique allowing quantitative visualization of living cells with subwavelength axial accuracy. Opt. Lett..

[20] Rappaz B., Charrière F., Depeursinge C., Magistretti P.J., Marquet P. (2008). Simultaneous cell morphometry and refractive index measurement with dual-wavelength digital holographic microscopy and dye-enhanced dispersion of perfusion medium. Opt. Lett..

[21] Boss D., Kühn J., Jourdain P., Depeursinge C., Magistretti P.J., Marquet P. (2013). Measurement of absolute cell volume, osmotic membrane water permeability, and refractive index of transmembrane water and solute flux by digital holographic microscopy. J. Biomed. Opt..

[22] Colomb T., Cuche E., Depeursinge C. (2005). Birefringence measurement by use of digital holographic microscopy: Examples with fiber optics and concrete samples. Optical Measurement Systems for Industrial Inspection Iv, Pts 1 and 2.

[23] Depeursinge C., Marquet P., Pavillon N., Boas D.A., Pitris C., Ramanujam N. (2011). Application of Digital Holographic Micros-copy in Biomedicine. Handbook of Biomedical Optics.

[24] Kastl L., Isbach M., Dirksen D., Schnekenburger J., Kemper B. (2017). Quantitative phase imaging for cell culture quality control. Cytometry A..

[25] Connor T.O., Rawat S., Markman A., Javidi B. (2018). Automatic cell identification and visualization using digital holographic microscopy with head mounted augmented reality devices. Appl. Opt..

[26] Rubin M., Stein O., Turko N.A., Nygate N., Roitshtain D., Karako L., Barnea I., Giryes R., Shaked N.T. (2019). TOP-GAN: Stain-free cancer cell classification using deep learning with a small training set. Med. Image Anal..

[27] Benzerdjeb N., Garbar C., Camparo P., Sevestre H. (2016). Digital Holographic Microscopy as Screening Tool for Cervical Cancer Preliminary Study. Cancer Cytopathol..

[28] Croft L.V., Mulders J.A., Richard D.J., Byrne K.O. (2019). Digital Holographic Imaging as a Method for Quantitative, Live Cell Imaging of Drug Response to Novel Targeted Cancer Therapies. Methods Mol. Biol..

[29] Kühn J., Shaffer E., Mena J., Breton B., Parent J., Rappaz B., Chambon M., Emery Y., Magistretti P., Depeursinge C. (2013). Label-free cytotoxicity screening assay by digital holographic microscopy. Assay Drug Dev. Technol..

[30] Barer R. (1953). Determination of dry mass, thickness, solid and water concentration in living cells. Nature.

[31] Krizova A., Collakova J., Dostal Z., Kvasnica L., Uhlirova H., Zikmund T., Vesely P., Chmelik R. (2015). Dynamic phase differences based on quantitative phase imaging for the objective evaluation of cell behavior. J. Biomed. Opt..

[32] Rappaz B., Cano E., Colomb T., Kühn J., Depeursinge C., Simanis V., Magistretti P.J., Marquet P. (2009). Noninvasive characterization of the fission yeast cell cycle by monitoring dry mass with digital holographic microscopy. J. Biomed. Opt..

[33] Cintora P., Arikkath J., Kandel M., Popescu G., Best-Popescu C. (2017). Cell Density Modulates Intracellular MassTransport in Neural Networks. Cytom. Part A.

[34] Di Caprio G., Ferrara M.A., Miccio L., Merola F., Memmolo P., Ferraro P., Coppola G. (2015). Holographic imaging of unlabelled sperm cells for semen analysis: A review. J. Biophotonics.

[35] Bianco V., Memmolo P., Leo M., Montresor S., Distante C., Paturzo M., Picart P., Javidi B., Ferraro P. (2018). Strategies for reducing speckle noise in digital holography. Light Sci. Appl..

[36] Pan F., Yang L., Xiao W. (2017). Coherent noise reduction in digital holographic microscopy by averaging multiple holograms recorded with a multimode laser. Opt. Express.

[37] Loiselle C.L., Bélanger E., Marquet P. (2020). Polychromatic digital holographic microscopy: A quasicoherent-noise-free imaging technique to explore the connectivity of living neuronal networks. Neurophotonics.

[38] Girshovitz P., Shaked N.T. (2012). Generalized cell morphological parameters based on interferometric phase microscopy and their application to cell life cycle characterization. Biomed. Opt. Express.

[39] Boss D., Hoffmann A., Rappaz B., Depeursinge C., Magistretti P.J., Van de Ville D., Marquet P. (2012). Spatially-resolved eigenmode decomposition of red blood cells membrane fluctuations questions the role of ATP in flickering. PLoS ONE.

[40] Eldridge W.J., Sheinfeld A., Rinehart M.T., Wax A. (2016). Imaging deformation of adherent cells due to shear stress using quantitative phase imaging. Opt. Lett..

[41] Lue N., Popescu G., Ikeda T., Dasari R.R., Badizadegan K., Feld M.S. (2006). Live cell refractometry using microfluidic devices. Opt. Lett..

[42] Kemper B., Kosmeier S., Langehanenberg P., Von Bally G., Bredebusch I., Domschke W., Schnekenburger J. (2007). Integral refractive index determination of living suspension cells by multifocus digital holographic phase contrast microscopy. J. Biomed. Opt..

[43] Curl C.L., Bellair C.J., Harris P.J., Allman B.E., Roberts A., Nugent K.A., Delbridge L.M. (2006). Single cell volume measurement by quantitative phase microscopy (QPM): A case study of erythrocyte morphology. Cell Physiol. Biochem..

[44] Bélanger E., Lévesque S.A., Rioux-Pellerin E., Lavergne P., Marquet P. (2019). Measuring Absolute Cell Volume Using Quantitative-Phase Digital Holographic Microscopy and a Low-Cost, Open-Source, and 3D-Printed Flow Chamber. Front. Phys..

[45] Rappaz B., Barbul A., Emery Y., Korenstein R., Depeursinge C., Magistretti P.J., Marquet P. (2008). Comparative study of human erythrocytes by digital holographic microscopy, confocal microscopy, and impedance volume analyzer. Cytometry. A.

[46] Pham T.A., Soubies E., Ayoub A., Lim J., Psaltis D., Unser M. (2020). Three-dimensional optical diffraction tomography with Lippmann Schwinger model. IEEE Trans. Comput. Imaging.

[47] Marian A., Charrière F., Colomb T., Montfort F., Kühn J., Marquet P., Depeursinge C. (2007). On the complex three-dimensional amplitude point spread function of lenses and microscope objectives: Theoretical aspects, simulations and measurements by digital holography. J. Microsc..

[48] Hillman T.R., Gutzler T., Alexandrov S.A., Sampson D.D. (2009). High-resolution, wide-field object reconstruction with synthetic aperture Fourier holographic optical microscopy. Opt. Express.

[49] Picazo J.A., Zalevsky Z., Garcia J., Mico V. (2017). Superresolved spatially multiplexed interferometric microscopy. Opt. Lett..

[50] Zheng C., Jin D., He Y., Lin H., Hu J., Yaqoob Z., So P.T.C., Zhou R. (2020). High spatial and temporal resolution synthetic aperture phase microscopy. Adv. Photonics.

[51] Kim M.K. (1999). Wavelength-scanning digital interference holography for optical section imaging. Opt. Lett..

[52] Montfort F., Colomb T., Charriere F., Kuhn J., Marquet P., Cuche E., Herminjard S., Depeursinge C. (2006). Submicrometer optical tomography by multiple-wavelength digital holographic microscopy. Appl. Opt..

[53] Kühn J., Montfort F., Colomb T., Rappaz B., Moratal C., Pavillon N., Marquet P., Depeursinge C. (2009). Submicrometer tomography of cells by multiple-wavelength digital holographic microscopy in reflection. Opt. Lett..

[54] Cuche E., Poscio P., Depeursinge C. (1997). Optical tomography by means of a numerical low-coherence holographic technique. J. Opt..

[55] Massatsch P., Charrière F., Cuche E., Marquet P., Depeursinge C. (2005). Time-domain optical coherence tomography with digital holographic microscopy. Appl. Opt..

[56] Delacrétaz Y., Pavillon N., Lang F., Depeursinge C. (2009). Off-axis low coherence interferometry contouring. Opt. Commun..

[57] Haeberle O., Belkebir K., Giovaninni H., Sentenac A. (2010). Tomographic diffractive microscopy: Basics, techniques and perspectives. J. Mod. Opt..

[58] Jin D., Zhou R.J., Yaqoob Z., So P.T.C. (2017). Tomographic phase microscopy: Principles and applications in bioimaging. J. Opt. Soc. Am. B.

[59] Lauer V. (2002). New approach to optical diffraction tomography yielding a vector equation of diffraction tomography and a novel tomographic microscope. J. Microsc..

[60] Charriere F., Marian A., Montfort F., Kuehn J., Colomb T., Cuche E., Marquet P., Depeursinge C. (2006). Cell refractive index tomography by digital holographic microscopy. Opt. Lett..

[61] Choi W., Fang-Yen C., Badizadegan K., Oh S., Lue N., Dasari R.R., Feld M.F. (2007). Tomographic phase microscopy. Nat. Methods.

[62] Debailleul M., Georges V., Simon B., Morin R., Haeberle O. (2009). High-resolution three-dimensional tomographic diffractive microscopy of transparent inorganic and biological samples. Opt. Lett..

[63] Soto J.M., Rodrigo J.A., Alieva T. (2018). Partially coherent illumination engineering for enhanced refractive index tomography. Opt. Lett..

[64] Chen X., Kandel M.E., Hu C., Lee Y.J., Popesu G. (2020). Wolf phase tomography (WPT) of transparent structures using partially coherent illumination. Light Sci. Appl..

[65] Cotte Y., Toy M.F., Pavillon N., Depeursinge C. (2010). Microscopy image resolution improvement by deconvolution of complex fields. Opt. Express.

[66] Sandoz P.A., Tremblay C., Van der Goot F.G., Frechin M. (2019). Image-based analysis of living mammalian cells using label-free 3D refractive index maps reveals new organelle dynamics and dry mass flux. PLoS Biol..

[67] Kandel M.E., He Y.R., Lee Y.J., Chen T.H.Y., Sullivan K.M., Aydin O., Saif M.T.A., Kong H., Sobh N., Popescu G. (2020). Phase imaging with computational specificity (PICS) for measuring dry mass changes in sub-cellular compartments. Nat. Commun..

[68] Guo R., Mirsky S.K., Barnea I., Dudaie M., Shaked N.T. (2020). Quantitative phase imaging by wide-field interferometry with variable shearing distance uncoupled from the off-axis angle. Opt. Express.

[69] Saleh B.E.A., Teich M.C. (2007). Fundamentals of Photonics.

[70] Hai N., Rosen J. (2019). Coded aperture correlation holographic microscope for single-shot quantitative phase and amplitude imaging with extended field of view. Opt. Express.

[71] Hai N., Rosen J. (2021). Single-plane and multiplane quantitative phase imaging by self-reference on-axis holography with phase-shifting method. Opt. Express.

[72] Hai N., Rosen J. Phase-contrast-based holographic quantitative phase imaging by only two exposures. Proceedings of the CLEO 2021.

[73] Hai N., Rosen J. (2020). Phase contrast-based phase retrieval: A bridge between qualitative phase contrast and quantitative phase imaging by phase retrieval algorithms. Opt. Lett..

[74] Juodkazis S., Nishimura K., Tanaka S., Misawa H., Gamaly E.G., Luther-Davies B., Hallo L., Nicolai P., Tikhonchuk V.T. (2006). Laser-induced microexplosion confined in the bulk of a sapphire crystal: Evidence of multimegabar pressures. Phys. Rev. Lett..

[75] Vailionis A., Gamaly E.G., Mizeikis V., Yang W., Rode A.V., Juodkazis S. (2011). Evidence of superdense aluminium synthesized by ultrafast microexplosion. Nat. Commun..

[76] Zhong L., Wang J., Sheng H., Zhang Z., Mao S.X. (2014). Formation of monatomic metallic glasses through ultrafast liquid quenching. Nature.

[77] Stoian R., Colombier J.P. (2020). Advances in ultrafast laser structuring of materials at the nanoscale. Nanophotonics.

[78] Hunter W.R. (1998). Measurement of Optical Constants in the Vacuum Ultraviolet Spectral Region.

[79] Hayasaki Y., Iwata K., Hasegawa S., Takita A., Juodkazis S. (2011). Time resolved axial-view of the dielectric breakdown under tight focusing in glass. Opt. Mater. Express.

[80] Hayasaki Y., Isaka M., Takita A., Hasegawa S., Juodkazis S. (2012). Photoacoustic sub-micrometer modifications of glass by pair of femtosecond laser pulses. Opt. Mater. Express.

[81] Hayasaki Y., Isaka M., Takita A., Juodkazis S. (2011). Time-resolved interferometry of femtosecond-laser induced processes under tight focusing and close-to optical breakdown inside borosilicate glass. Opt. Express.

[82] Hayasaki Y., Fukuda S.I., Hasegawa S., Juodkazis S. (2017). Two-color pump-probe interferometry of ultra-fast light-matter interaction. Sci. Rep..

[83] Gamaly E.G., Rode A.V. (2018). Ultrafast re-structuring of the electronic landscape of transparent dielectrics: New material states (Die-Met). Appl. Phys. A.

[84] Shao Z., Cao X., Luo H., Jin P. (2018). Recent progress in the phase-transition mechanism and modulation of vanadium dioxide materials. NPG Asia Mater..

[85] Makarov S.V., Zalogina A.S., Tajik M., Zuev D.A., Rybin M.V., Kuchmizhak A.A., Juodkazis S., Kivshar Y. (2017). Light-Induced Tuning and Reconfiguration of Nanophotonic Structures. Laser Photonics Rev..

[86] Sun Q., Jiang H.B., Liu Y., Wu Z.X., Yang H., Gong Q.H. (2016). Diagnose parameters of plasma induced by femtosecond laser pulse in quartz and glasses. Front. Phys. China.

[87] Ryu M., Honda R., Balčytis A., Vongsvivut J., Tobin M.J., Juodkazis S., Morikawa J. (2019). Hyperspectral mapping of anisotropy. Nanoscale Horiz..

[88] Anand V., Ng S.H., Katkus T., Juodkazis S. (2021). Spatio-Spectral-Temporal Imaging of Fast Transient Phenomena Using a Random Array of Pinholes. Adv. Photonics Res..

[89] Anand V., Katkus T., Linklater D.P., Ivanova E.P., Juodkazis S. (2020). Lensless Three-Dimensional Quantitative Phase Imaging Using Phase Retrieval Algorithm. J. Imaging.

[90] Gabor D.A. (1948). New microscopic principle. Nature.

[91] Carter W.H. (1970). Computational reconstruction of scattering objects from holograms. J. Opt. Soc. Am. A.

[92] Fercher A., Bartelt H., Becker H., Wiltschko E. (1979). Image formation by inversion of scattered field data: Experiments and computational simulation. Appl. Opt..

[93] Park C., Lee K., Baek Y., Park Y. (2020). Low-coherence optical diffraction tomography using a ferroelectric liquid crystal spatial light modulator. Opt. Express.

[94] Kuś A., Krauze W., Kujawińska M. (2015). Active limited-angle tomographic phase microscope. J. Biomed. Opt..

[95] Park Y., Diez-Silva M., Popescu G., Lykotrafitis G., Choi W., Feld M.S., Suresh S. (2008). Refractive index maps and membrane dynamics of human red blood cells parasitized by Plasmodium falciparum. Proc. Natl. Acad. Sci. USA.

[96] Oh J., Ryu J.S., Lee M., Jung J., Han S., Chung H.J., Park Y. (2020). Three-dimensional label-free observation of individual bacteria upon antibiotic treatment using optical diffraction tomography. Biomed. Opt. Express.

[97] Devaney A. (1981). Inverse-scattering theory within the Rytov approximation. Opt. Lett..

[98] Hugonnet H., Kim Y.W., Lee M., Shin S., Hruban R.H., Hong S.-M., Park Y. (2021). Multiscale label-free volumetric holographic histopathology of thick-tissue slides with subcellular resolution. Adv. Photonics.

[99] Gustafsson M.G., Shao L., Carlton P.M., Wang C.R., Golubovskaya I.N., Cande W.Z., Agard D.A., Sedat J.W. (2008). Three-dimensional resolution doubling in wide-field fluorescence microscopy by structured illumination. Biophys. J..

[100] Shin S., Kim D., Kim K., Park Y. (2018). Super-resolution three-dimensional fluorescence and optical diffraction tomography of live cells using structured illumination generated by a digital micromirror device. Sci. Rep..

[101] Barer R., Tkaczyk S. (1954). Refractive index of concentrated protein solutions. Nature.

[102] Kamilov U.S., Papadopoulos I.N., Shoreh M.H., Goy A., Vonesch C., Unser M., Psaltis D. (2015). Learning approach to optical tomography. Optica.

[103] Chen M., Ren D., Liu H.Y., Chowdhury S., Waller L. (2020). Multi-layer Born multiple-scattering model for 3D phase microscopy. Optica.

[104] Liu H.Y., Liu D., Mansour H., Boufounos P.T., Waller L., Kamilov U.S. (2017). SEAGLE: Sparsity-driven image reconstruction under multiple scattering. IEEE Trans. Comput. Imaging.

[105] Baek Y., Lee K., Shin S., Park Y. (2019). Kramers–Kronig holographic imaging for high-space-bandwidth product. Optica.

[106] Baek Y., Park Y. (2021). Intensity-based holographic imaging via space-domain Kramers–Kronig relations. Nat. Photonics.

[107] Horstmeyer R., Chung J., Ou X., Zheng G., Yang C. (2016). Diffraction tomography with Fourier ptychography. Optica.

[108] Li J., Matlock A.C., Li Y., Chen Q., Zuo C., Tian L. (2019). High-speed in vitro intensity diffraction tomography. Adv. Photonics.

[109] Shin S., Kim K., Yoon J., Park Y. (2015). Active illumination using a digital micromirror device for quantitative phase imaging. Opt. Lett..

[110] Lee K., Kim K., Kim G., Shin S., Park Y. (2017). Time-multiplexed structured illumination using a DMD for optical diffraction tomography. Opt. Lett..

[111] Soto J.M., Rodrigo J.A., Alieva T. (2017). Label-free quantitative 3D tomographic imaging for partially coherent light microscopy. Opt. Express.

[112] Ryu D., Ryu D., Baek Y., Cho H., Kim G., Kim Y.S., Lee Y., Kim Y., Ye J.C., Min H.S. (2020). DeepRegularizer: Rapid Resolution Enhancement of Tomographic Imaging using Deep Learning. IEEE Trans. Biomed. Eng..

[113] Kim G., Ahn D., Kang M., Jo Y., Ryu D., Kim H., Song J., Ryu J.S., Choi G., Chung H.J. (2019). Rapid and label-free identification of individual bacterial pathogens exploiting three-dimensional quantitative phase imaging and deep learning. bioRxiv.

[114] Lee M., Lee Y.H., Song J., Kim G., Jo Y., Min H., Kim C.H., Park Y.J.E. (2020). Deep-learning-based three-dimensional label-free tracking and analysis of immunological synapses of CAR-T cells. eLife.

[115] Jo Y., Cho H., Park W.S., Kim G., Ryu D., Kim Y.S., Lee M., Joo H., Jo H., Lee S. (2020). Data-driven multiplexed microtomography of endogenous subcellular dynamics. bioRxiv.

[116] Kim E.H., Park S., Kim Y.K., Moon M., Park J., Lee K.J., Lee S., Kim Y.P. (2020). Self-luminescent photodynamic therapy using breast cancer targeted proteins. Sci. Adv..

[117] Park S., Ahn J.W., Jo Y., Kang H.Y., Kim H.J., Cheon Y., Kim J.W., Park Y., Lee S., Park K. (2020). Label-free tomographic imaging of lipid droplets in foam cells for machine-learning-assisted therapeutic evaluation of targeted nanodrugs. ACS Nano.

[118] Esposito M., Fang C., Cook K.C., Park N., Wei Y., Spadazzi C., Bracha D., Gunaratna R.T., Laevsky G., DeCoste C.J. (2021). TGF-β-induced DACT1 biomolecular condensates repress Wnt signalling to promote bone metastasis. Nat. Cell Biol..

[119] Kim K., Yoon J., Park Y. (2015). Simultaneous 3D visualization and position tracking of optically trapped particles using optical diffraction tomography. Optica.

[120] Kim K., Park Y. (2017). Tomographic active optical trapping of arbitrarily shaped objects by exploiting 3D refractive index maps. Nat. Commun..

[121] Chatterjee A., Sanchez J.A.C., Yamauchi T., Taupin V., Couvrette J., Gorodetsky A.A. (2020). Cephalopod-inspired optical engineering of human cells. Nat. Commun..

[122] Kak A.C., Slaney M. (1988). Principles of Computerized Tomographic Imaging.

[123] Kou S.S., Sheppard C.J.R. (2008). Image formation in holographic tomography. Opt. Lett..

[124] Liu P.Y., Chin L.K., Ser W., Chen H.F., Hsieh C.M., Lee C.H., Sung K.B., Ayi T.C., Yap P.H., Liedberg B. (2016). Cell refractive index for cell biology and disease diagnosis: Past, present and future. Lab Chip.

[125] Balasubramani V., Tu H.Y., Lai X.J., Cheng C.J. (2019). Adaptive wavefront correction structured illumination holographic tomography. Sci. Rep..

[126] Vertu S., Yamada I., Delaunay J.J., Haeberlé O., Flüge J. (2009). Diffraction microtomography with sample rotation: Primary result on the influence of a missing apple core in the recorded frequency space. Proc. SPIE.

[127] Kostencka J., Kozacki T., Kuś A., Kujawińska M. (2015). Accurate approach to capillary-supported optical diffraction tomography. Opt. Express.

[128] Lin Y.C., Chen H.C., Tu H.Y., Liu C.Y., Cheng C.J. (2017). Optically driven full-angle sample rotation for tomographic imaging in digital holographic microscopy. Opt. Lett..

[129] Balasubramani V., Anand V., Rai M.R., Rosen J., Cheng C.J., Minin O.V., Minin I.V. (2020). Binary square axicon with chiral focusing properties for optical trapping. Opt. Eng..

[130] Balasubramani V., Lai X.J., Lin Y.C., Cheng C.J. (2018). Integrated dual-tomography for refractive index analysis of free-floating single living cell with isotropic superresolution. Sci. Rep..

[131] Cheng C.J., Chien K.C.C., Lin Y.C. (2018). Digital hologram for data augmentation in learning-based pattern classification. Opt. Lett..

[132] Balasubramani V., Tu H.Y., Haung H.C., Cheng C.J. (2020). All-optical dual-tomography for free-floating live cell imaging and analysis. Imaging and Applied Optics Congress.

[133] Balasubramani V., Montresor S., Tu H.Y., Huang C.H., Picart P., Cheng C.J. (2021). Influence of noise-reduction techniques in sparse-data sample rotation tomographic imaging. App. Opt..

[134] Schürmann M., Cojoc G., Girardo S., Ulbricht E., Guck J., Muller P. (2017). Three-dimensional correlative single-cell imaging utilizing fluorescence and refractive index tomography. J. Biophotonics.

[135] Tahara T., Quan X., Otani R., Takaki Y., Matoba O. (2018). Digital holography and its multidimensional imaging applications: A review. Microscopy.

[136] Liu C., Malek M., Poon I., Jiang L., Siddiquee A.M., Sheppard C.J.R., Roberts A., Quiney H., Zhang D., Yuan X. (2019). Simultaneous dual-contrast three-dimensional imaging in live cells via optical diffraction tomography and fluorescence. Photonics Res..

[137] Quan X., Nitta K., Matoba O., Xia P., and Awatsuji Y. (2015). Phase and fluorescence imaging by combination of digital holographic microscopy and fluorescence microscopy. Opt. Rev..

[138] Dudenkova V.V., Zakharov Y.N. (2016). Multimodal combinational holographic and fluorescence fluctuation microscopy to obtain spatial super-resolution. J. Phys. Conf. Ser..

[139] Smolyanskaya O.A., Lazareva E.N., Nalegaev S.S., Petrov N.V., Zaytsev K.I., Timoshina P.A., Tuchina D.K., Toropova Y.G., Kornyushin O.V., Babenko A.Y. (2019). Multimodal Optical Diagnostics of Glycated Biological Tissues. Biochemistry.

[140] Matoba O., Quan X., Xia P., Awatsuji Y., Nomura T. (2017). Multimodal Imaging Based on Digital Holography. Proc. IEEE.

[141] Zernike F. (1955). How I discovered phase contrast. Science.

[142] Goodman J.W., Lawrence R.W. (1967). Digital image formation from electronically detected holograms. Appl. Phys. Lett..

[143] Van Rooij J., Kalkman J. (2019). Large scale high sensitivity optical diffraction tomography of zebrafish. Biomed. Opt. Express.

[144] Kim K., Yoon J., Park Y.K. (2016). Large-scale optical diffraction tomography for inspection of optical plastic lenses. Opt. Lett..

[145] Ma L., Wang H., Li Y., Zhang H. (2009). Elimination of zero-order diffraction and conjugate image in off-axis digital holography. J. Mod. Opt..

[146] Kostencka J., Kozacki T., Józwik M. (2017). Holographic tomography with object rotation and two-directional off-axis illumination. Opt. Express.

[147] Simon B., Debailleul M., Houkal M., Ecoffet C., Bailleul J., Lambert J., Spangenberg A., Liu H., Soppera O., Haeberlé O. (2017). Tomographic diffractive microscopy with isotropic resolution. Optica.

[148] Lim J., Lee K., Jin K.H., Shin S., Lee S., Park Y., Ye J.C. (2015). Comparative study of iterative reconstruction algorithms for missing cone problems in optical diffraction tomography. Opt. Express.

[149] Hosseini P., Zhou R., Kim Y.-H., Peres C., Diaspro A., Kuang C., Yaqoob Z., So P.T.C. (2016). Pushing phase and amplitude sensitivity limits in interferometric microscopy. Opt. Lett..

[150] Kostencka J., Kozacki T., Dudek M., Kujawińska M. (2014). Noise suppressed optical diffraction tomography with autofocus correction. Opt. Express.

[151] Van Rooij J., Kalkman J. (2020). Polarization contrast optical diffraction tomography. Biomed. Opt. Express.

[152] Saba A., Lim J., Ayoub A.B., Antoine E.E., Psaltis D. (2021). Polarization-sensitive optical diffraction tomography. Optica.

[153] Menzel M., Axer M., Amunts K., De Raedt H., Michielsen K. (2019). Diattenuation Imaging reveals different brain tissue properties. Sci. Rep..

[154] Choi H., Li Z., Jeong K., Zuponcic J., Ximenes E., Turek J., Ladisch M., Nolte D.D. (2021). Phase-sensitive intracellular Doppler fluctuation spectroscopy. Phys. Rev. Appl..

[155] Jo Y., Cho H., Lee S.Y., Choi G., Kim G., Min H.-S., Park Y.K. (2019). Quantitative Phase Imaging and Artificial Intelligence*:* A Review. IEEE J. Sel. Top. Quantum Electron..

[156] Shu X., Sansare S., Jin D., Zeng X., Tong K.-Y., Pandey R., Zhou R. (2021). Artificial-Intelligence-Enabled Reagent-Free Imaging Hematology Analyzer. Adv. Intell. Syst..

[157] Gómez V.H., Herazo E.L., Smith S.T. (2019). X-ray computed tomography: From medical imaging to dimensional metrology. Precis. Eng..

[158] Ferrucci M., Leach R.K., Giusca C., Carmignato S., Dewulf W. (2015). Towards geometrical calibration of x-ray computed tomography systems—A review. Meas. Sci. Technol..

[159] Filippou V., Tsoumpas C. (2018). Recent advances on the development of phantoms using 3D printing for imaging with CT, MRI, PET, SPECT, and ultrasound. Med. Phys..

[160] Kujawińska M., Krauze W., Baczewska M., Kuś A., Ziemczonok M. Comparative study of laboratory and commercial limited-angle holographic tomography setups. Proceedings of the SPIE 10887, Quantitative Phase Imaging V.

[161] Ziemczonok M., Kuś A., Wasylczyk P., Kujawińska M. (2019). 3D-printed biological cell phantom for testing 3D quantitative phase imaging systems. Sci. Rep..

[162] Ziemczonok M., Kuś A.T., Kujawinska M. Quantifying the performance of holographic tomography systems using the 3D-printed biological cell phantom. Proceedings of the SPIE 11249, Quantitative Phase Imaging VI.

[163] LaFratta C., Baldacchini T. (2017). Two-Photon Polymerization Metrology: Characterization Methods of Mechanisms and Microstructures. Micromachines.

[164] Huang Z., Chi-Pong Tsui G., Deng Y., Tang C.-Y. (2020). Two-photon polymerization nanolithography technology for fabrication of stimulus-responsive micro/nano-structures for biomedical applications. Nanotechnol. Rev..

[165] Park C., Shin S., Park Y. (2018). Generalized quantification of three-dimensional resolution in optical diffraction tomography using the projection of maximal spatial bandwidths. J. Opt. Soc. Am. A.

[166] Horstmeyer R., Heintzmann R., Popescu G., Waller L., Yang C. (2016). Standardizing the resolution claims for coherent microscopy. Nat. Photonics.

[167] Huang J., Bao Y., Gaylord T.K. (2020). Three-dimensional phase optical transfer function in axially symmetric microscopic quantitative phase imaging. J. Opt. Soc. Am. A.

[168] De Groot P.J. (2021). The instrument transfer function for optical measurements of surface topography. J. Phys. Photonics.

[169] Waller L. (2020). Physics-constrained computational imaging. Emerging Topics in Artificial Intelligence 2020.

[170] Belthangady C., Royer L.A. (2019). Applications, promises, and pitfalls of deep learning for fluorescence image reconstruction. Nat. Methods.

[171] McCann M.T., Jin K.H., Unser M. (2017). Convolutional neural networks for inverse problems in imaging: A review. IEEE Signal Process. Mag..

[172] Rivenson Y., Wu Y., Ozcan A. (2019). Deep learning in holography and coherent imaging. Light Sci. Appl..

[173] Bostan E., Heckel R., Chen M., Kellman M., Waller L. (2020). Deep phase decoder: Self-calibrating phase microscopy with an untrained deep neural network. Optica.

[174] Tian L., Waller L. (2015). 3D intensity and phase imaging from light field measurements in an LED array microscope. Optica.

[175] Pierré W., Hervé L., Allier C., Morales S., Grudinin S., Chowdhury S., Dhellemmes M. (2021). Deep learning framework applied to optical diffraction tomography (ODT). Three-Dimensional and Multidimensional Microscopy: Image Acquisition and Processing.

[176] Li J., Mengu D., Yardimci N.T., Luo Y., Li X., Veli M., Ozcan A. (2021). Spectrally encoded single-pixel machine vision using diffractive networks. Sci. Adv..

[177] Pinkard H., Stuurman N., Ivanov I.E., Anthony N.M., Ouyang W., Li B., Waller L. (2021). Pycro-Manager: Open-source software for customized and reproducible microscope control. Nat. Methods.

[178] Tomocube (2021). http://www.tomocube.com.

[179] Lyncee Tec (2021). https://www.lynceetec.com.

[180] Nanolive (2021). https://www.nanolive.com.

